# Effects of shading, fertilization, and irrigation on floral display and honey yield in *Agastache rugosa* in controlled pot culture

**DOI:** 10.3389/fpls.2025.1679318

**Published:** 2025-12-16

**Authors:** Ji-Min Park, Young-Ki Kim, Hyun-Jun Kim, Sung-Joon Na

**Affiliations:** 1 Special Forest Resources Division, National Institute of Forest Science, Suwon, Republic of Korea; 2 Department of Forest Resources, Sunchon National University, Suncheon, Republic of Korea

**Keywords:** *Agastache rugosa*, nectar secretion, honey production potential, floral productivity, shade treatment, watering interval, nutrient supply

## Abstract

Sustainable cultivation of *Agastache rugosa* requires quantitative guidance on how to balance ornamental floral display with nectar-mediated honey production under realistic limitations in light, nutrients, and water. Although this species is widely recognized as both an ornamental and melliferous plant, integrated management strategies that jointly optimize floral traits and honey yield under combined abiotic treatments remain poorly defined. This study aimed to clarify how shading, fertilization, and irrigation interact to shape growth, flowering, nectar traits, and estimated honey yield in *A. rugosa*, and to identify cultivation combinations that simultaneously support landscape quality and nectar provision. A three-factor, three-level factorial experiment was conducted in a randomized split-plot design with shading at 0, 35, and 55%, fertilization with N:P:K 20:20:20 at 0, 1, and 3 g/L, and irrigation at 2-, 4-, and 6-day intervals. Measured response variables included plant height, number of inflorescences per plant, inflorescence length, number of flowers per inflorescence, total flower number per plant, nectar volume per flower, and free sugar content per volume. Estimated honey yield per plant was calculated from nectar sugar content and flower number using a standard conversion approach. Fertilization and irrigation produced the largest increases in plant size, floral output, and estimated honey yield across shading levels, whereas nectar volume and sugar concentration were numerically stable and showed no consistent treatment response. The highest estimated honey yield, 0.7722 g/plant, occurred under 35% shading with 1 g/L fertilization and 2-day irrigation, where moderate shading and frequent watering enhanced floral display without diluting nectar sugar content. Together, these results indicate that moderate resource inputs can decouple floral architecture from nectar quality, enabling dual-purpose management of *A. rugosa* as an ornamental and melliferous crop in climate-adaptive production systems.

## 1 Introduction

Evidence has been presented from a number of regions indicating that climate extremes, including drought, heat and irregular rainfall, have the capacity to influence plant-pollinator interactions and honey production in agricultural systems (Olsen et al., 2022; Díaz-Calafat et al., 2025; Frigero et al., 2025). Reduced floral availability and changes in nectar secretion and sugar concentration alter foraging behavior and hive productivity, directly affecting apicultural value and ecosystem services (Descamps et al., 2021a, Descamps et al., 2021b; Fernandes et al., 2023; Plos et al., 2023; Oliveira et al., 2025). These pressures have increased the demand for climate-resilient nectariferous crops that can sustain pollinator health and honey yields in working landscapes and green spaces (Zhang et al., 2022; Plos et al., 2023).

Multifunctional agriculture targets crop that provides combined benefits, including ornamental display and melliferous resources (McGrath et al., 2021; Akplo et al., 2023; Plos et al., 2023). Managing this dual role is challenging because floral productivity and nectar quality often respond differently to light, nutrients, and water (Nepi et al., 2001; Plos et al., 2023). Floral display primarily governs pollinator attraction and visitation, whereas nectar volume and sugar concentration determine per-visit rewards and foraging persistence. Consequently, honey production is controlled by both the number of flowers and the nectar available per flower, yet flower abundance does not always scale with nectar output or composition, so extrapolation from single floral metrics can be misleading (Burquez and Corbet, 1991; Petanidou, 2007).

*Agastache rugosa* is a perennial Lamiaceae species valued for aromatic foliage, conspicuous inflorescences, and abundant nectar (Zielińska and Matkowski, 2014; Horga et al., 2024). Nectaries at the base of the corolla tube facilitate pollinator access and visitation within the genus (Roy et al., 2017). The species is widely used in East Asia for culinary and pharmacological purposes and has gained interest as a melliferous crop in Korea due to reliable late-season flowering and nectar secretion (Nechita et al., 2023; Hong et al., 2020; Nam et al., 2020). Its occurrence in mesic habitats indicates tolerance to partial shade and moderate soil moisture, and recent work reports substantial physiological plasticity along light and nutrient gradients (Rural Development Administration (RDA), 2018; Wang et al., 2023; Rosli et al., 2024a, Rosli et al., 2024b; Rosli et al., 2025a, Rosli et al., 2025b).

Previous studies have examined the effects of single environmental factors such as light, fertilization, or water on growth performance and essential oil content in related systems, yet joint controls of multiple abiotic inputs on floral display and nectar traits remain insufficiently quantified for *A. rugosa* (Plos et al., 2023; Mansinhos et al., 2024; Göttlinger et al., 2025). This gap is critical when the management goal is to maintain landscape value while securing stable nectar provision and honey yield. In Lamiaceae, nectar traits are strongly influenced by physiological and microclimatic conditions and may vary independently of flower number, so simple assumptions that more flowers necessarily translate into greater honey production are not always valid (Burquez and Corbet, 1991; Petanidou, 2007). These considerations motivate an integrative test of how light, nutrient supply, and water availability together shape growth, floral display, nectar traits, and their implications for honey production within a single experimental framework (Barberis et al., 2022; Brito Vera and Pérez, 2024; Mansinhos et al., 2024).

The central question of this study is how combined shading, fertilization, and irrigation influence floral and nectar traits in *A. rugosa*, and why these responses matter for dual-use cultivation. We addressed this question using a three-factor, three-level factorial experiment under controlled conditions to identify management combinations that support both ornamental productivity and melliferous performance.

## 2 Materials and methods

### 2.1 Plant material

Seedlings were propagated in April 2021 from a single bulked seed lot collected from healthy, phenotypically uniform *A. rugosa* plants cultivated at the experimental station in Suwon, South Korea during autumn 2020. The seeds were air-dried, cleaned, and stored in paper envelopes at 4 °C in the dark for approximately six months until use. All plants were grown in 2.4 L pots filled with a standardized soil mixture of peat moss, perlite, and vermiculite (5:3:2, v/v/v). Seedlings were acclimated for one month in a greenhouse at the National Institute of Forest Science (37°16′N, 127°00′E, 48 m a.s.l., Suwon, South Korea) under standard environmental control. During acclimation, plants received natural sunlight. Experimental treatments were implemented from June to late September 2021, following standardized cultivation protocols for medicinal herbs in Korea (Rural Development Administration (RDA), 2018).

### 2.2 Experimental design

**Figure 1:**
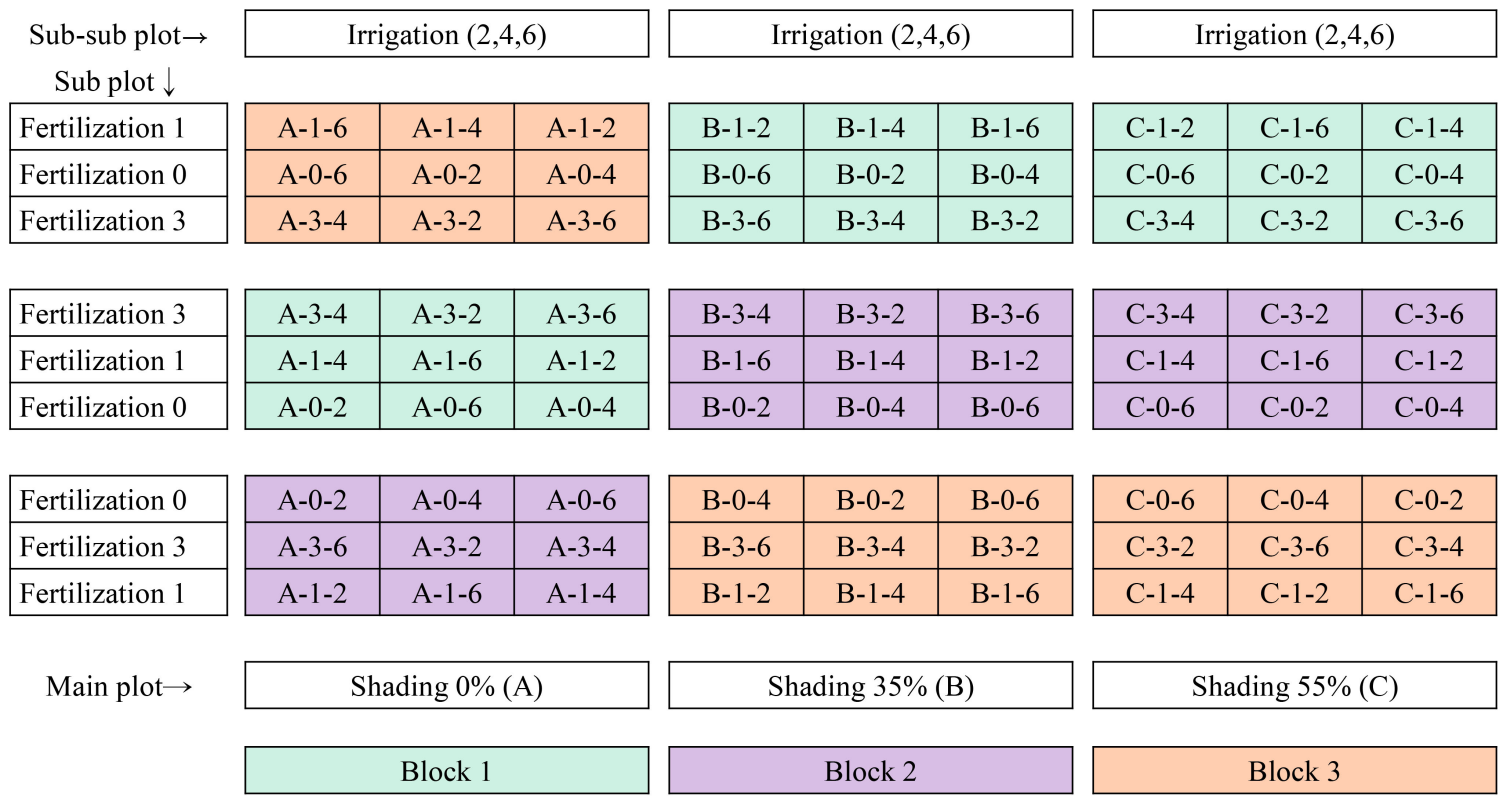
Experimental layout of the three-factor, three-level factorial experiment applied to *A. rugosa* using a split-split-plot design under a randomized complete block structure. The main plots represent shading treatments (0%, 35%, 55%), the subplots correspond to fertilization levels (0, 1, 3 g/L), and the sub-subplots refer to irrigation intervals (2-, 4-day, 6-day). This factorial arrangement produced 27 treatment combinations. For clarity, treatments are described throughout the manuscript by their factor levels (e.g., 35% shading + 1 g/L fertilization + four-day irrigation) rather than by abstract codes.

factorial field experiment was used to evaluate the interactive effects of shading, fertilization, and irrigation on the growth and nectar traits of *A. rugosa*. The study followed a split–split-plot arrangement within a randomized complete block design (RCBD) with three levels per factor and three blocks, yielding 81 experimental units (3³ × 3). Shading was assigned to main plots, fertilization to subplots, and irrigation interval to sub-subplots (**Figure 1**). Shade structures targeted nominal reductions of 0, 35, and 55% based on manufacturer specifications. Transmittance was checked at canopy height with a quantum sensor to confirm that treatments reduced incoming light in the intended order. Because continuous light logging was not implemented, we report relative transmittance among treatments rather than absolute irradiance.

A balanced water-soluble fertilizer (Multifeed N-P-K ratio of 20–20–20, Haifa Chemicals Co., Haifa Bay, Israel) was applied at 0 g/L (no fertilization), 1 g/L (moderate fertilization), and 3 g/L (high fertilization). These levels were chosen based on preliminary trials and local recommendations for medicinal herbs, which indicated that 1 g/L is near the lower range of optimal growth while 3 g/L approaches the threshold beyond which no further yield benefit is expected. Fertilizer solution was applied every 12 days, with 200 mL per plant at each application.

Irrigation intervals of 2, 4, and 6 days were selected to represent frequent, moderate, and reduced watering regimes under container cultivation. Each plant received 200 mL of water at every irrigation event, resulting in similar total water volumes across treatments over a 12-day cycle. Irrigation and fertilization were implemented as independent factors; when fertilizer application coincided with an irrigation event, the fertilizer solution was included within the 200 mL volume to maintain equal total liquid input among treatments. All irrigation and fertilization were completed before 11:00 a.m. to minimize evaporative loss and diurnal variability.

Within each block, main plots (shading) were randomly assigned to positions, and subplots (fertilization) and sub-subplots (irrigation) were randomized within their respective higher-level plots. Border rows were included to reduce edge effects, and plots were separated by buffer zones to limit lateral shading and water movement.

### 2.3 Nectar sampling and honey yield estimation

To secure sufficient nectar volume and ensure consistency in floral age, nectar sampling followed a repeated procedure based on floral lifespan labeling. On Day 1, all open flowers were removed from the target inflorescences. On Day 2, newly opened flowers were labeled with red tags, and on Day 3, flowers opening on that day were labeled with blue tags. On Day 4, all blue-labeled flowers, corresponding to Day 2 of their floral lifespan, were harvested for nectar analysis. This protocol standardized nectar collection to flowers of comparable physiological age and minimized variation related to floral senescence. For each of the 27 treatment combinations, six biological replicates were sampled per phase, each consisting of pooled nectar from 8–10 flowers collected from one plant, resulting in 80 flowers per treatment per phase. The procedure was conducted twice during the peak flowering period (8–15 September), representing early- and mid-bloom phases. The late bloom phase was not sampled because nectar secretion was highly irregular and often below detection limits; we note that this exclusion may cause our estimates to be conservative for total season-long nectar yield.

All nectar sampling was carried out between 14:00 and 16:00 p.m. on rain-free days at air temperatures of 20–26°C to reduce diurnal and weather-related variability in nectar production. Nectar was extracted using the centrifugation method described by Na et al. (2024); Park et al. (2025), which is suitable for small-flowered species such as *A. rugosa*. Immediately after collection, nectar samples were stored at −20°C until chemical analysis.

Free sugar content was analyzed using high-performance liquid chromatography (HPLC; Dionex Ultimate 3000, Dionex, Sunnyvale, CA, USA). Deionized water served as the mobile phase at a flow rate of 0.5 mL min−1−1, and the column oven was maintained at 80°C. Detection was carried out with a Shodex RI-101 refractive index detector (Showa Denko, New York, NY, USA) coupled to an Aminex 87P column (300 × 7.8 mm, Bio-Rad, Hercules, CA, USA). Free sugar concentration was determined by the external standard method, using high-purity (99.5%) sucrose, glucose, and fructose (Sigma-Aldrich, St. Louis, MO, USA) to construct calibration curves that bracketed the range of expected sample concentrations. Each analytical batch included solvent blanks and quality-control standards to monitor baseline drift and carry-over; batches that did not meet pre-defined acceptance criteria were re-run.


$$\text{Estimated Honey yield }\left(\text{g}/\text{plant}\right)=\text{Nectar sugar content }{\left(\text{mg}/\text{flower}\right)}^{1}\times {\text{Number of flowersperplant}}^{2}\times 1.15^{3}\times 0.001\left(\text{for unit conversion}:\text{mg to g}\right)$$

^1^ Nectar sugar content (μg/flower) = Nectar volume (μL/flower) × Free Sugar content (μg/μL).

^2^ Number of flowers per plant (ea/plant): Determined based on plant growth metrics.

^3^ Honey potential = sugar content: honey = 85:100 (Petanidou, 2003).

Honey yield per plant was estimated using established formulas integrating nectar volume (µL/flower), free sugar content (µg/µL), nectar sugar content (mg/flower), and number of flowers (ea/plant), as previously described for quantifying floral resource availability (Hicks et al., 2016; Pamminger et al., 2019; Na et al., 2024, Na et al., 2025).

### 2.4 Statistical analyses

To examine relationships among morphological traits, nectar traits, Pearson’s correlation coefficients were calculated for all measured and derived variables. This analysis was used to assess potential multicollinearity among traits and to identify variables most closely associated with estimated honey yield, following recent work on trait integration and floral resource allocation (Vannette, 2020; Plos et al., 2023; Malovrh et al., 2024b).

Because many response variables were correlated, we first used multivariate analysis of variance (MANOVA) as a multivariate screen to test the overall effects of shading, fertilization, irrigation, and their interactions on a core set of primary traits (plant height, number of inflorescences, inflorescence length, number of flowers per inflorescence, nectar volume per flower, and free sugar content). Derived variables such as nectar sugar content and estimated honey yield were excluded from the MANOVA to avoid redundancy. MANOVA was chosen to account for covariance among traits and to control the experiment-wise Type I error rate when evaluating multiple responses simultaneously (Fründ et al., 2010).

We then fitted three-way ANOVA models for each individual trait. This sequence provides a clear link from an integrated test of joint responses across traits to trait-level summaries, while limiting the number of *post hoc* contrasts. Terms that were not significant at the multivariate level were summarized descriptively without inferential claims.

For each ANOVA, when a main effect or interaction was significant (*p* < 0.05), least squares means were compared using Tukey’s honest significant difference (HSD) test to control the family-wise error rate (Yousefzadeh et al., 2022; Plos et al., 2023; Xing et al., 2023). When interactions were significant, we examined simple-effect LSM with Tukey adjustment for multiple comparisons; when interactions were not significant, we reported level-wise LSM without assigning alphabetical groupings and restricted interpretation to main effects. Estimated marginal means are presented with standard errors or 95% confidence intervals. All analyses were conducted in JMP Pro version 18.2.0 (SAS Institute Inc., Cary, NC, USA), with the significance threshold set at *p* < 0.05.

## 3 Results

### 3.1 Morphological and nectar-related trait variation among treatments

The morphological and nectar traits of *A. rugosa* varied across the 27 combinations of shading, fertilization, and irrigation treatments (**Tables 1**, **2**). Plant height ranged from 86.5 cm under 35 % shading, 0 g/L fertilization, and 6-day irrigation to 130.0 cm under 35 % shading, 3 g/L fertilization, and 4-day irrigation. The number of inflorescences per plant ranged from 2.22 to 10.80. Inflorescence length ranged from 4.07 cm to 9.37 cm, and the number of flowers per inflorescence ranged from 57 to 260. The total number of flowers/plant ranged from 201 to 1,608 across treatments.

**Table 1:** Morphological traits of *A. rugosa* under combinations of shading (0%, 35%, 55%), fertilization (0, 1, 3 g/L), and irrigation interval (2-, 4-, 6-days).

Treatment			Plant height (cm)		No. of inflorescences (ea/plant)		Inflorescence length (cm)		No. of flowers (ea/inflorescence)		No. of flowers (ea/plant)	
Shading	Fertilization	Irrigation	Mean	SD	Mean	SD	Mean	SD	Mean	SD	Mean	SD
0%	non	2day	122	8.79	4.08	1.68	7.35	1.74	155	51.6	672	390
		4day	120	19	3.18	1.08	5.35	1.09	123	23.6	384	121
		6day	112	8.73	4.27	2.20	5.31	1.13	115	27.6	485	223
	1g/L	2day	123	18.2	5.58	2.23	9.37	3.67	260	122	1,545	951
		4day	101	21.0	5.30	1.77	6.40	1.40	150	70.2	858	536
		6day	114	9.56	5.33	3.16	6.10	1.26	113	31.9	638	415
	3g/L	2day	121	15.3	10.6	3.83	6.52	1.65	149	52.1	1,556	698
		4day	117	9.39	6.83	2.21	5.56	1.51	113	45.4	804	425
		6day	121	15.4	8.33	3.52	5.53	1.90	123	50.3	1,068	552
35%	non	2day	115	14.1	3.56	1.81	4.95	1.24	98.3	54	323	197
		4day	109	13.6	2.22	1.72	5.93	2.04	115	80.6	201	138
		6day	86.5	24.7	5.18	3.40	4.07	1.55	57	29	298	239
	1g/L	2day	119	9.91	8.45	3.59	6.12	1.11	139	63.2	1,247	935
		4day	118	13.1	7.17	2.37	5.03	1.66	104	48.8	754	494
		6day	115	22.8	7.75	4.22	5.39	1.84	95.3	38.6	867	754
	3g/L	2day	118	15.7	10.8	4.37	5.88	1.79	136	54.8	1,531	1071
		4day	130	5.25	8.75	3.31	5.37	1.42	100	39.5	858	413
		6day	113	11.8	9.11	4.40	5.04	1.72	80.6	56	829	776
55%	non	2day	118	16.0	3.78	2.17	6.05	1.55	132	63.4	507	407
		4day	115	11.4	2.82	1.33	5.08	1.87	86	55.3	276	274
		6day	97.5	16.8	5.42	1.98	5.13	1.41	112	55	606	337
	1g/L	2day	107	19.8	8.45	2.73	6.03	1.48	116	46.3	1,000	520
		4day	113	20.8	6.40	2.67	4.66	1.30	79.8	46.2	488	281
		6day	121	11.8	4.92	2.19	5.94	2.18	112	63.3	537	399
	3g/L	2day	123	15.7	7.60	3.10	7.33	2.03	196	69.1	1,608	969
		4day	117	5.34	6.00	2.58	5.46	1.43	134	47.1	869	613
		6day	111	21.0	7.17	2.72	5.64	0.71	130	31.9	977	525

Mean, the average; SD, standard deviation. Each value represents the mean of three replicates. Parameters include plant height (cm), number of inflorescences each per plant, inflorescence length (cm), number of flowers each per inflorescence, and total number of flowers each per plant.

**Table 2:** Nectar traits and estimated honey yield of *A. rugosa* under varying shading, fertilization, and irrigation treatments.

Treatment			Nectar volume (µL/flower)		Free sugar content (µg/µL)		Nectar sugar content (mg/flower)		Honey yield (g/plant)	
Shading	Fertilization	Irrigation	Mean	SD	Mean	SD	Mean	SD	Mean	SD
0%	non	2day	0.42	0.10	626	200	0.26	0.08	0.21	0.11
		4day	0.44	0.10	896	66.2	0.39	0.08	0.18	0.04
		6day	0.40	0.11	728	192	0.28	0.07	0.13	0.08
	1g/L	2day	0.42	0.08	881	314	0.35	0.10	0.47	0.38
		4day	0.39	0.08	886	94.8	0.34	0.05	0.37	0.26
		6day	0.37	0.06	746	323	0.28	0.12	0.24	0.13
	3g/L	2day	0.31	0.11	853	130	0.27	0.12	0.50	0.25
		4day	0.32	0.04	860	316	0.28	0.11	0.25	0.22
		6day	0.33	0.10	832	328	0.26	0.11	0.25	0.17
35%	non	2day	0.42	0.08	802	236	0.33	0.08	0.1	0.07
		4day	0.38	0.12	909	146	0.35	0.12	0.08	0.06
		6day	0.37	0.08	842	216	0.31	0.09	0.09	0.12
	1g/L	2day	0.39	0.10	888	170	0.34	0.09	0.77	0.72
		4day	0.37	0.17	809	157	0.31	0.17	0.31	0.23
		6day	0.40	0.08	809	315	0.31	0.11	0.37	0.20
	3g/L	2day	0.39	0.16	711	385	0.24	0.06	0.42	0.44
		4day	0.23	0.08	796	209	0.19	0.09	0.16	0.08
		6day	0.30	0.08	952	292	0.27	0.07	0.20	0.24
55%	non	2day	0.36	0.12	666	225	0.25	0.13	0.15	0.12
		4day	0.37	0.06	770	340	0.28	0.11	0.06	0.07
		6day	0.31	0.05	897	228	0.27	0.04	0.16	0.12
	1g/L	2day	0.43	0.10	850	193	0.36	0.10	0.45	0.32
		4day	0.42	0.11	789	281	0.32	0.11	0.22	0.17
		6day	0.31	0.08	837	366	0.25	0.12	0.11	0.10
	3g/L	2day	0.34	0.11	901	192	0.30	0.08	0.57	0.33
		4day	0.29	0.10	899	194	0.27	0.12	0.24	0.24
		6day	0.22	0.10	589	254	0.13	0.06	0.17	0.19

Mean, the average; SD, standard deviation. Measured parameters include nectar volume per flower (µL), free sugar content (µg/µL), and derived nectar sugar content (mg). Estimated honey yield per plant (g) were calculated based on flower count and sugar content per flower. Values represent means from three replicates per treatment.

Nectar traits also showed measurable ranges. Nectar volume per flower varied from 0.22 to 0.44 µL, and free sugar content varied from 589 to 952 µg/µL. Combining these parameters, nectar sugar content per flower ranged from 0.13 to 0.39 mg. Using nectar sugar content per flower and flower number per plant, the estimated honey yield per plant ranged from 0.06 to 0.77 g/plant, which corresponds to 4.95–62.1 kg/ha when scaled to field density. The maximum estimated honey yield (0.77 g/plant, 62.1 kg/ha) occurred at 35 % shading, 1 g/L fertilization, and 2-day irrigation.

### 3.2 Trait interrelationships revealed by correlation analysis

**Figure 2:**
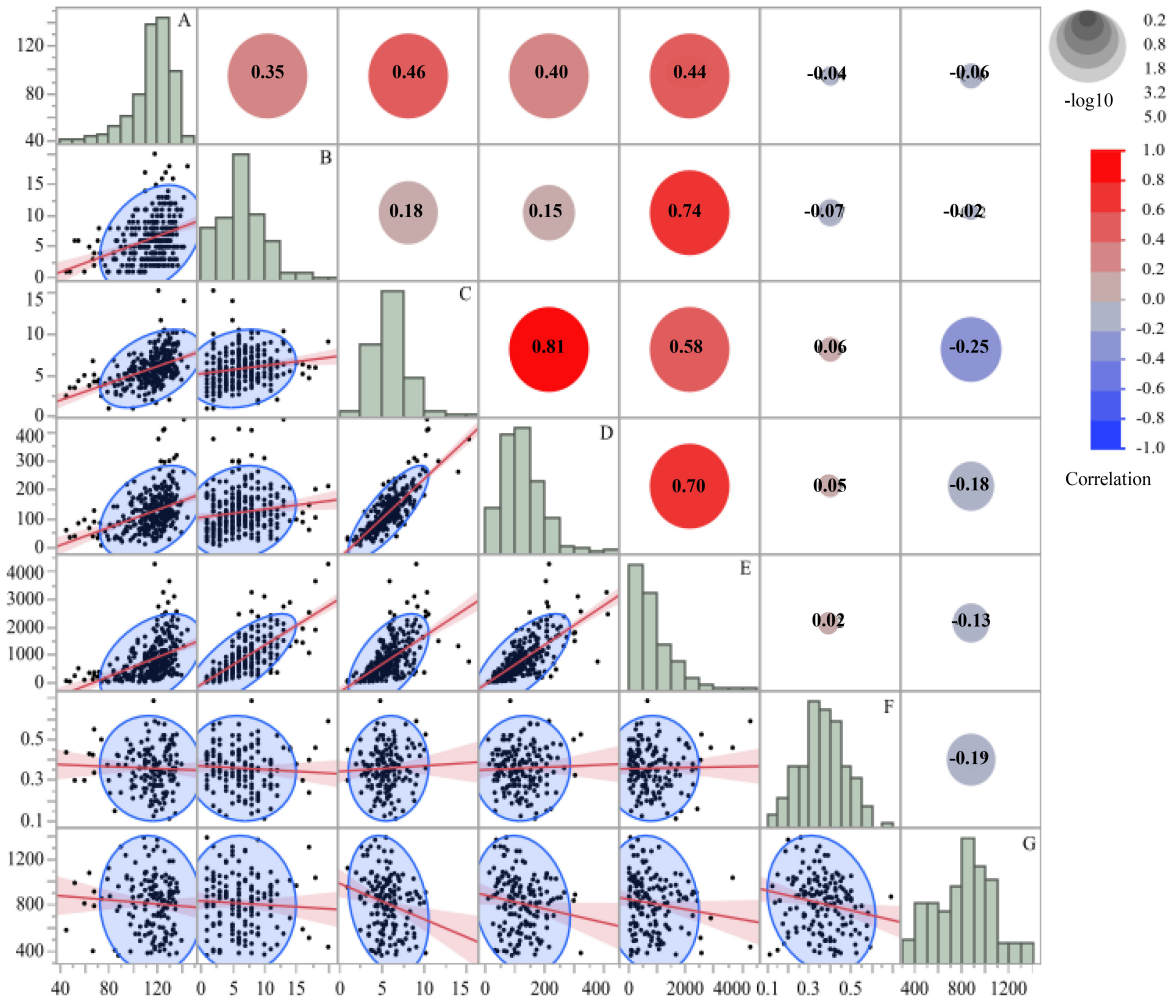
Correlation matrix of seven morphological and nectar traits in *A. rugosa*, including: **(A)** plant height (cm), **(B)** number of inflorescences per plant, **(C)** inflorescence length (cm), **(D)** number of flowers per inflorescence, **(E)** total number of flowers per plant, **(F)** nectar volume per flower (µL), and **(G)** free sugar content (µg/µL). Pearson coefficients (r) are shown in the upper triangle, with scatterplots and regression lines in the lower triangle. Histograms represent trait distributions along the diagonal.

Pearson correlation analysis was conducted across all samples to quantify interrelationships among seven morphological and nectar traits of *A. rugosa* (**Figure 2**). The traits included plant height (cm), inflorescences per plant, inflorescence length (cm), flowers per inflorescence, total flowers per plant, nectar volume per flower (µL), and free sugar content (µg/µL).

Plant height was positively correlated with inflorescences per plant (r = 0.35, *p* < 0.001) and inflorescence length (r = 0.46, *p* < 0.001). Inflorescence length showed positive correlations with flowers per inflorescence (r = 0.58, *p* < 0.001) and total flowers per plant (r = 0.81, *p* < 0.001). Flowers per inflorescence was also positively correlated with total flowers per plant (r = 0.70, *p* < 0.001).

Nectar traits showed little association with morphological traits. Correlation coefficients between nectar volume per flower or free sugar content and any morphological trait were all |r|< 0.07. The correlation between nectar volume per flower and free sugar content was r = −0.19. Thus, floral display traits were positively associated with one another, whereas nectar volume and sugar concentration varied largely independently of plant size and flower number.

### 3.3 Multifactorial impacts of shading, fertilization, and irrigation on traits

Morphological and reproductive traits of *A. rugosa* were strongly influenced by fertilization and irrigation, whereas nectar traits showed little response to the tested environmental factors (**Table 3**). MANOVA indicated significant main effects of shading (Wilks’ Lambda = 0.787, F = 2.67, *p* < 0.01; Pillai’s Trace = 0.217, F = 2.58, *p* < 0.01), fertilization (Wilks’ Lambda = 0.606, F = 5.97, *p* < 0.001; Pillai’s Trace = 0.409, F = 5.45, *p* < 0.001), and irrigation (Wilks’ Lambda = 0.704, F = 4.03, *p* < 0.001; Pillai’s Trace = 0.318, F = 4.01, *p* < 0.001) on the set of primary traits. Among interaction terms, shading × fertilization and fertilization × irrigation were significant in MANOVA, whereas shading × irrigation and the three-way interaction were not (*p* > 0.05).

**Table 3:** MANOVA results showing the effects of shading, fertilization, and irrigation, as well as their interactions, on the morphological and nectar traits of *A. rugosa*.

Source	DF	Wilks’ Lambda		Pillai’s Trace	
		Value	F-value (*p*-value)	Value	F-value (*p*-value)
Shading	12	0.787	2.67^**^	0.217	2.58^**^
Fertilization	12	0.606	5.97^***^	0.409	5.45^***^
Irrigation	12	0.704	4.03^***^	0.318	4.01^***^
Shading x Fertilization	24	0.709	1.91^**^	0.320	1.87^**^
Shading x Irrigation	24	0.837	0.96^ns^	0.172	0.96^ns^
Fertilization x Irrigation	24	0.615	2.75^***^	0.428	2.58^***^
Shading x Fertilization x Irrigation	48	0.697	0.988^ns^	0.338	0.977^ns^

ns, non-significant; **p < 0.01; ***p < 0.001. Wilks’ Lambda, and Pillai’s Trace statistics are reported with corresponding F-values and significance levels. Degrees of freedom (DF) are based on treatment levels and trait variables.

Across combinations of shading, fertilization, and irrigation, *A. rugosa* expressed a wide range of phenotypes (**Supplementary Table 1**). Under higher fertilization and shorter irrigation intervals, plant height approached 128.2–129.4 cm and flowers per inflorescence reached 198.2 at 55 % shading, 3 g/L fertilization, and 2-day irrigation. Based on MANOVA supported main effect contrasts of least squares means, the 2-day irrigation interval increased plant height by 5.7 % and total flowers per plant by 77.2 % relative to the 6-day interval. Relative to 0 g/L, fertilization at 1 g/L and 3 g/L increased inflorescences/plant by 97.5 % and 130.1 % and increased total flowers per plant by 147.4 % and 192.0 %, respectively.

Shading modified inflorescence architecture. At the main effect level, 0 % shading produced 38.9 % more flowers per inflorescence than 35 % shading and 19.7 % more than 55 % shading, whereas the greatest plant heights and flower counts were often associated with minimal or moderate shading. Inflorescence length and flowers/inflorescence showed treatment-specific maxima within shading levels (**Supplementary Table 1**).

Nectar traits were comparatively stable across treatments. Nectar volume per flower ranged from 0.30 to 0.42 µL and free sugar content ranged from 625 to 951 µg/µL, with no significant effects of shading, fertilization, irrigation, or their interactions detected in MANOVA or subsequent univariate tests.

### 3.4 Distinct and interactive effects of abiotic treatments on morphological and nectar traits

Three-way ANOVA showed that fertilization and irrigation had significant main effects on all morphological traits of *A. rugosa* (**Table 4**). Shading had significant main effects on all traits except plant height and total flowers per plant.

**Table 4:** Three-way ANOVA results for the morphological traits of *A. rugosa* in response to shading, fertilization, and irrigation treatments and their interactions.

Trait	Treatment	N	DF	SS	*F* (*p*-value)
Plant height	Shading	2	2	817.2	1.915^ns^
	Fertilization	2	2	2,973	6.967^***^
	Irrigation	2	2	2,833	6.639^***^
	Shading x Fertilization	4	4	4,503	5.276^**^
	Shading x Irrigation	4	4	1,519	2.719^*^
	Fertilization x Irrigation	4	4	6,387	7.484^***^
	Shading x Fertilization x Irrigation	8	8	3,782	2.216^ns^
No. of inflorescences (ea/plant)	Shading	2	2	79.18	4.927^*^
	Fertilization	2	2	999.6	62.19^***^
	Irrigation	2	2	123.5	7.684^***^
	Shading x Fertilization	4	4	125.6	3.908^*^
	Shading x Irrigation	4	4	3.752	0.117^ns^
	Fertilization x Irrigation	4	4	97.25	3.025^**^
	Shading x Fertilization x Irrigation	8	8	65.60	1.020^ns^
Inflorescence Length (cm)	Shading	2	2	58.23	9.893^***^
	Fertilization	2	2	20.10	3.415^*^
	Irrigation	2	2	98.23	16.69^***^
	Shading x Fertilization	4	4	34.86	2.962^*^
	Shading x Irrigation	4	4	36.09	3.067^*^
	Fertilization x Irrigation	4	4	13.23	1.124^ns^
	Shading x Fertilization x Irrigation	8	8	30.03	1.276^ns^
No. of Flowers (ea/Inflorescence)	Shading	2	2	84,640	13.57^**^
	Fertilization	2	2	23,019	3.691^*^
	Irrigation	2	2	136,874	21.95^***^
	Shading x Fertilization	4	4	75,892	6.085^***^
	Shading x Irrigation	4	4	32,276	2.588^ns^
	Fertilization x Irrigation	4	4	14,049	1.126^ns^
	Shading x Fertilization x Irrigation	8	8	58,555	2.347^**^
No. of Flowers (ea/plant)	Shading	2	2	1,017,043	1.546^ns^
	Fertilization	2	2	24,647,002	37.47^***^
	Irrigation	2	2	13,632,863	20.73^***^
	Shading x Fertilization	4	4	2,269,780	1.726^*^
	Shading x Irrigation	4	4	443,073	0.337^ns^
	Fertilization x Irrigation	4	4	3,813,358	2.899^**^
	Shading x Fertilization x Irrigation	8	8	1,043,854	0.397^ns^

ns, non-significant; *p < 0.05; **p < 0.01; ***p < 0.001. Traits include plant height (cm), number of inflorescences each per plant, inflorescence length (cm), number of flowers each per inflorescence, and total number of flowers each per plant.

Interaction effects were trait-specific. The shading × fertilization interaction was significant for plant height, inflorescences per plant, inflorescence length, flowers per inflorescence, and total flowers per plant. The shading × irrigation interaction was significant for plant height and inflorescence length, but not for inflorescences per plant, flowers per inflorescence, or total flowers per plant. The fertilization × irrigation interaction was significant for plant height, inflorescences per plant, and total flowers per plant, and not significant for inflorescence length or flowers per inflorescence. The three-way interaction (shading × fertilization × irrigation) was significant only for flowers per inflorescence and not significant for the other morphological traits.

For nectar traits, nectar volume per flower differed only among fertilization levels, while shading, irrigation, and all interaction terms were not significant (**Table 5**). Free sugar content did not differ for any main effect or interaction.

**Table 5:** Three-way ANOVA results for the nectar traits of *A. rugosa*, specifically nectar volume per flower (µL) and free sugar content (µg/µL), in response to shading, fertilization, and irrigation treatments and their interactions. No interaction terms were significant for nectar traits.

Characteristic	Source	N	DF	SS	*F* (*p*-value)
Nectar Volume (µL/flower)	Shading	2	2	0.036	1.859^ns^
	Fertilization	2	2	0.235	12.26^***^
	Irrigation	2	2	0.068	3.550^ns^
	Shading x Fertilization	4	4	0.021	0.548^ns^
	Shading x Irrigation	4	4	0.072	1.885^ns^
	Fertilization x Irrigation	4	4	0.019	0.494^ns^
	Shading x Fertilization x Irrigation	8	8	0.038	0.498^ns^
Free Sugar Content (µg/µL)	Shading	2	2	33,668	0.275^ns^
	Fertilization	2	2	45,104	0.368^ns^
	Irrigation	2	2	74,390	0.607^ns^
	Shading x Fertilization	4	4	88,720	0.362^ns^
	Shading x Irrigation	4	4	113,382	0.463^ns^
	Fertilization x Irrigation	4	4	258,277	1.055^ns^
	Shading x Fertilization x Irrigation	8	8	626,712	1.279^ns^

ns, non-significant; ***p < 0.001.

### 3.5 Trait-wise least squares means under shading, fertilization, and irrigation

**Figure 3:**
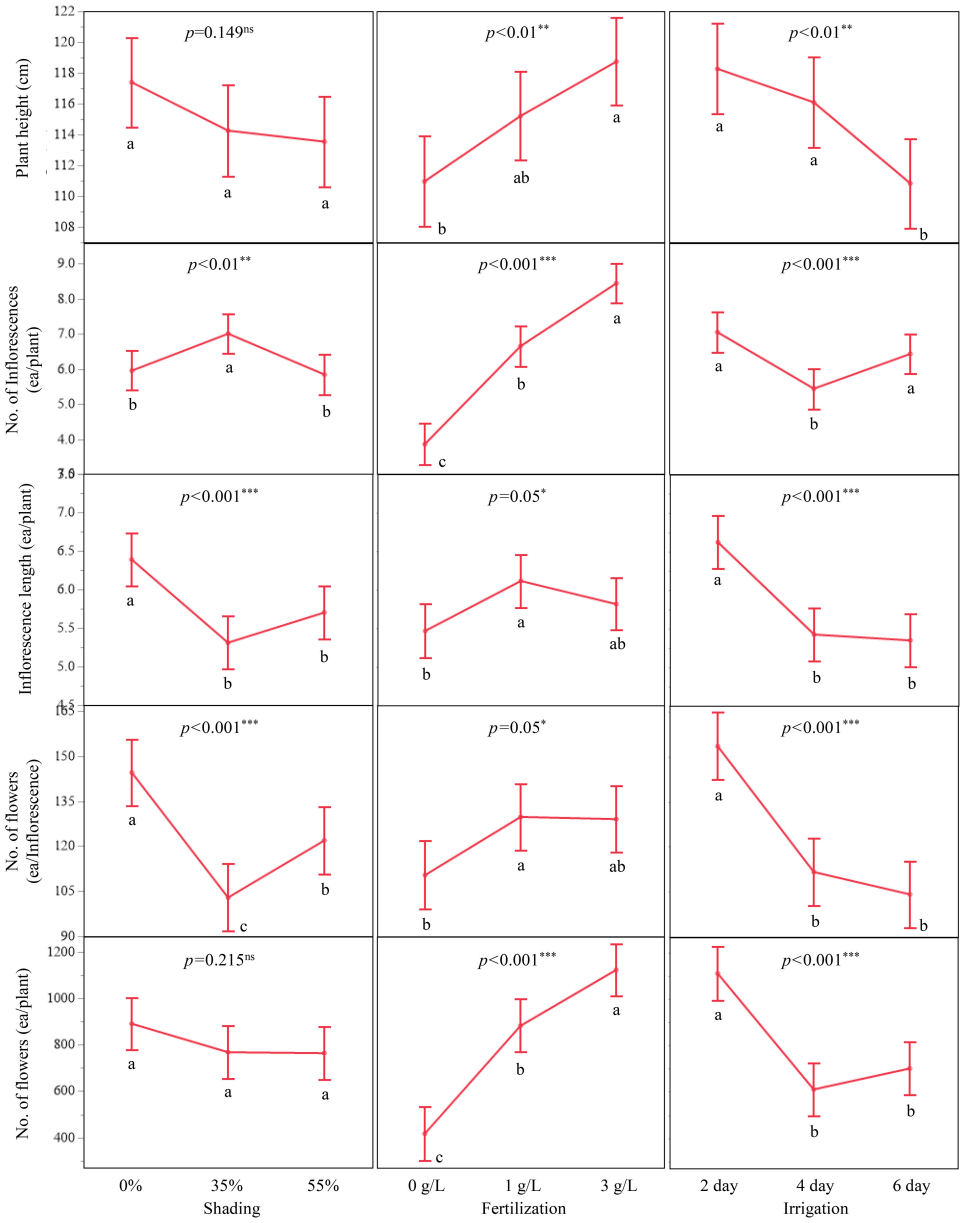
Least squares mean (LSM) plots showing the main effects of shading (0%, 35%, 55%), fertilization (0, 1, 3 g/L), and irrigation interval (2-, 4-, 6-day) on morphological traits of *A. rugosa*. Traits include plant height (cm), number of inflorescences per plant, inflorescence length (cm), number of flowers per inflorescence, and total number of flowers per plant. Error bars indicate standard errors, and letters denote Tukey HSD groupings (*p<* 0.05).

Least squares means from the ANOVA showed clear responses of growth and floral traits to the fertilization and irrigation treatments (**Figure 3**; **Supplementary Table 2**). Plant height increased along the fertilization gradient from 110.43 ± 1.60 cm at 0 g/L to 114.62 ± 1.57 cm at 1 g/L and 118.95 ± 1.53 cm at 3 g/L. Across the irrigation treatments, height was 118.48 ± 1.57 cm at a 2-day interval, 115.51 ± 1.58 cm at a 4-day interval, and 110.01 ± 1.55 cm at a 6-day interval. When plants were grouped by shading treatment, mean heights were 116.82 ± 1.56 cm at 0 %, 113.43 ± 1.58 cm at 35 %, and 113.75 ± 1.57 cm at 55 % shading.

Specific treatment combinations produced higher values than the main-treatment averages. For example, 3 g/L combined with 2-day irrigation and 3 g/L combined with 4-day irrigation gave heights of 121.23 ± 2.62 cm and 121.33 ± 2.67 cm, respectively, and 0 % shading with 2-day irrigation resulted in 121.84 ± 2.62 cm (**Supplementary Figure 1**). The combination of 35 % shading, 3 g/L fertilization, and 4-day irrigation yielded a maximum height of 130.08 ± 4.48 cm (**Supplementary Table 2**).

The number of inflorescences per plant increased from 3.83 ± 0.29 at 0 g/L to 6.60 ± 0.29 at 1 g/L and 8.46 ± 0.28 at 3 g/L. At the irrigation level with the highest mean, plants watered every 2 days produced 7.10 ± 0.29 inflorescences per plant. The combination of 3 g/L and 2-day irrigation gave 9.99 ± 0.48 inflorescences/plant (**Supplementary Figure 1**), and 35 % shading with 3 g/L and 2-day irrigation produced 10.83 ± 0.82 inflorescences/plant. Among the shading treatments, 35 % produced the largest mean number of inflorescences per plant (7.00 ± 0.29; **Supplementary Table 2**).

Inflorescence length also increased under higher nutrient supply and shorter irrigation intervals. At 0 % shading with 2-day irrigation, the mean inflorescence length was 7.75 ± 0.29 cm. The longest inflorescences, 9.37 ± 0.50 cm, were observed at 0 % shading with 1 g/L fertilization and 2-day irrigation (**Supplementary Table 2**).

Flowers per inflorescence increased from 110.46 ± 6.13 at 0 g/L to 129.85 ± 6.00 at 1 g/L and 134.50 ± 5.87 at 3 g/L. The combination of 3 g/L with 2-day irrigation yielded 175.19 ± 10.05 flowers per inflorescence, and 0 % shading with 1 g/L and 2-day irrigation produced 259.69 ± 17.15 flowers per inflorescence (**Supplementary Table 2**).

Total flowers per plant followed similar patterns. 416.86 ± 68.86 at 0 g/L to 881.58 ± 67.35 at 1 g/L and 1,206.68 ± 65.90 at 3 g/L nutrient supply. The combination of 3 g/L with 2-day irrigation resulted in 1 808.92 ± 112.80 flowers per plant, and 55 % shading with 3 g/L and 2-day irrigation produced the highest observed value of 2,339.78 ± 192.50 flowers per plant (**Supplementary Table 1**).

**Figure 4:**
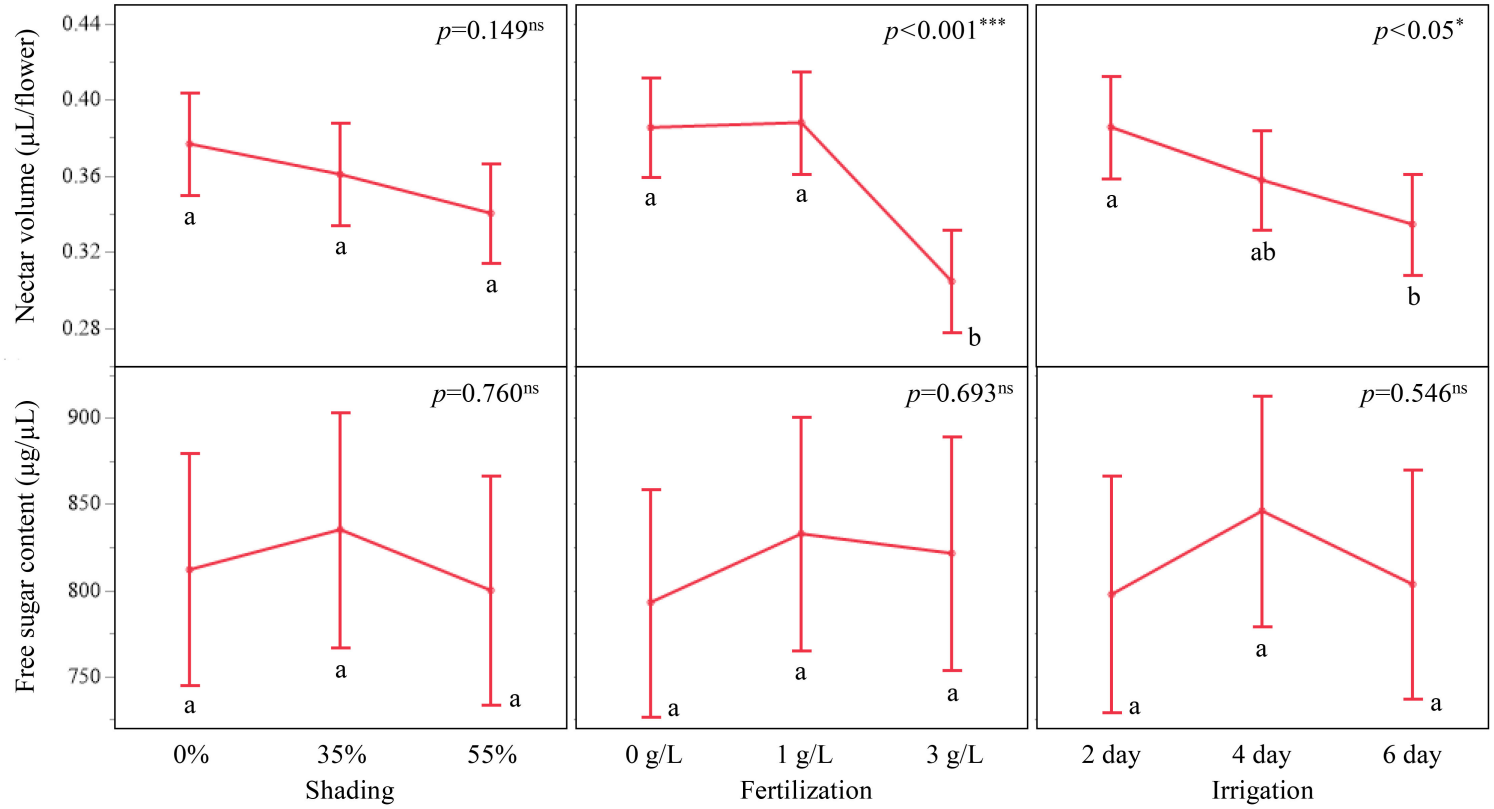
Least squares mean (LSM) plots of nectar volume (µL/flower) and free sugar content (µg/µL) in response to shading, fertilization, and irrigation treatments in *A. rugosa*. While fertilization and irrigation significantly influenced nectar volume, no treatment had a statistically significant effect on free sugar content. Error bars represent SE; different letters indicate Tukey HSD groups (*p* < 0.05).

Nectar traits varied over a smaller range than the morphological traits (**Figure 4**; **Supplementary Figure 2**). Nectar volume per flower was 0.43 ± 0.03 µL at 0 g/L, 0.39 ± 0.03 µL at 1 g/L, and 0.30 ± 0.03 µL at 3 g/L. A volume of 0.549 ± 0.053 µL was recorded at 0 g/L combined with 2-day irrigation, and 0.871 ± 0.092 µL at 0 % shading with 0 g/L and 2-day irrigation (**Supplementary Table 2**).

Free sugar content (µg/µL) showed modest differences among treatments. for example, 897.33 ± 50.01 µg/µL at 35 % shading and 797.57 ± 50.93 µg/µL at 2-day irrigation. Across all combinations, free sugar content per volume ranged from 625.67 ± 147.90 µg/µL at 0 % shading with 0 g/L and 2-day irrigation to 1,510.83 ± 147.90 µg/µL at 35 % shading with 3 g/L and 6-day irrigation (**Supplementary Table 2**).

### 3.6 Effects of environmental factors on estimated honey yield (g/plant)

The analysis of estimated honey yield per plant was used to evaluate how shading, fertilization, and irrigation influenced nectar-derived production under the experimental conditions. The linear model explained 36.1 % of the variance in honey yield (F = 2.89, *p* < 0.01; R² = 0.361, adjusted R² = 0.236). In this model, fertilization and irrigation had significant effects on honey yield, whereas shading and all interaction terms were not significant (**Table 6**).

**Table 6:** Summary of three-way ANOVA results for estimated honey yield (g/plant) in *A. rugosa*. The model includes main effects and interactions among shading, fertilization, and irrigation.

Source	DF	SS	MS	F (*p*-value)
Shading	2	0.08326		0.7249^ns^
Fertilization	2	1.634		14.23^***^
Irrigation	2	1.474		12.84^***^
Shading x Fertilization	4	0.4725		2.057^ns^
Shading x Irrigation	4	0.09434		0.4107^ns^
Fertilization x Irrigation	4	0.5357		2.332^ns^
Shading x Fertilization x Irrigation	8	0.2067		0.4500^ns^
Model	26	4.316	0.1660	2.890^***^
Error	133	7.638	0.5743	–

ns, not significant; ***p < 0.001.

Honey yield per plant increased with fertilizer concentration up to 1 g/L and then declined slightly at 3 g/L. Least squares means were 0.1298 g/plant at 0 g/L, 0.3667 g/plant at 1 g/L, and 0.3076 g/plant at 3 g/L (F = 14.23, *p* < 0.001). Tukey-adjusted comparisons indicated that 1 g/L produced significantly higher honey yield than both 0 g/L and 3 g/L. Irrigation frequency also affected honey yield (F = 12.84, *p* < 0.001). 0.4046 g/plant at a 2-day interval, 0.2081 g/plant at a 4-day interval, and 0.1911 g/plant at a 6-day interval, with the 2-day interval differing significantly from both longer irrigation intervals. Shading showed no significant effect on estimated honey yield (F = 0.72, *p* = 0.486). least squares means were 0.2899 g/plant at 0 %, 0.2952 g/plant at 35 %, and 0.2365 g/plant at 55 % shading.

**Figure 5:**
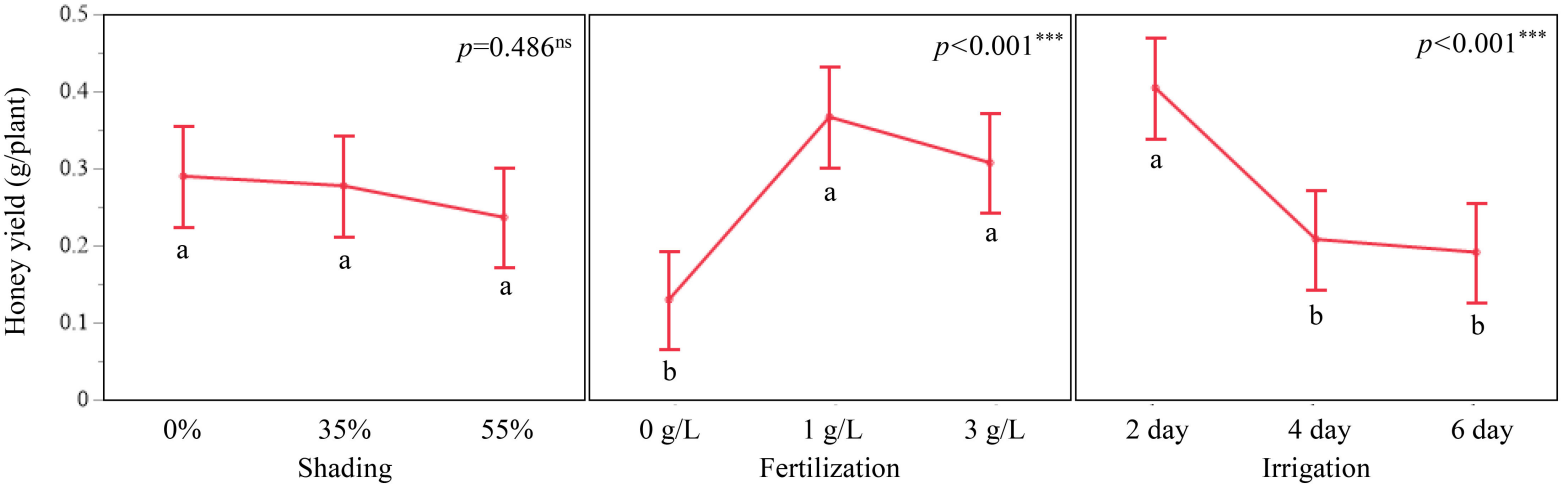
Main effect plots of shading, fertilization, and irrigation on estimated honey yield (g/plant) in *A. rugosa*. Bars represent LSM ± SE; letters indicate significant differences based on Tukey’s HSD (*p* < 0.05).

**Figure 6:**
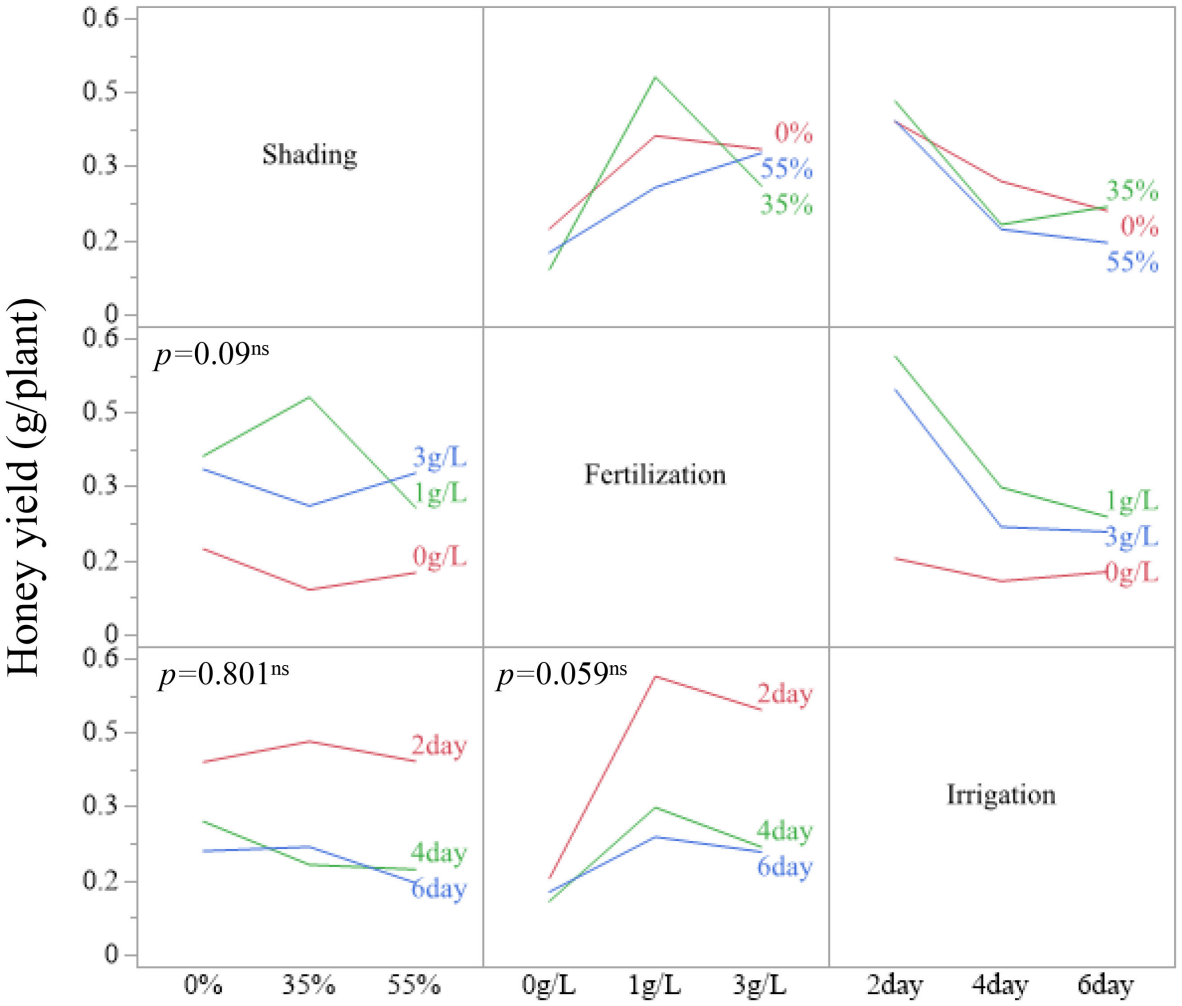
Two-way interaction plots of LSM for estimated honey yield in *A. rugosa* across shading, fertilization, and irrigation treatments.

**Figure 7:**
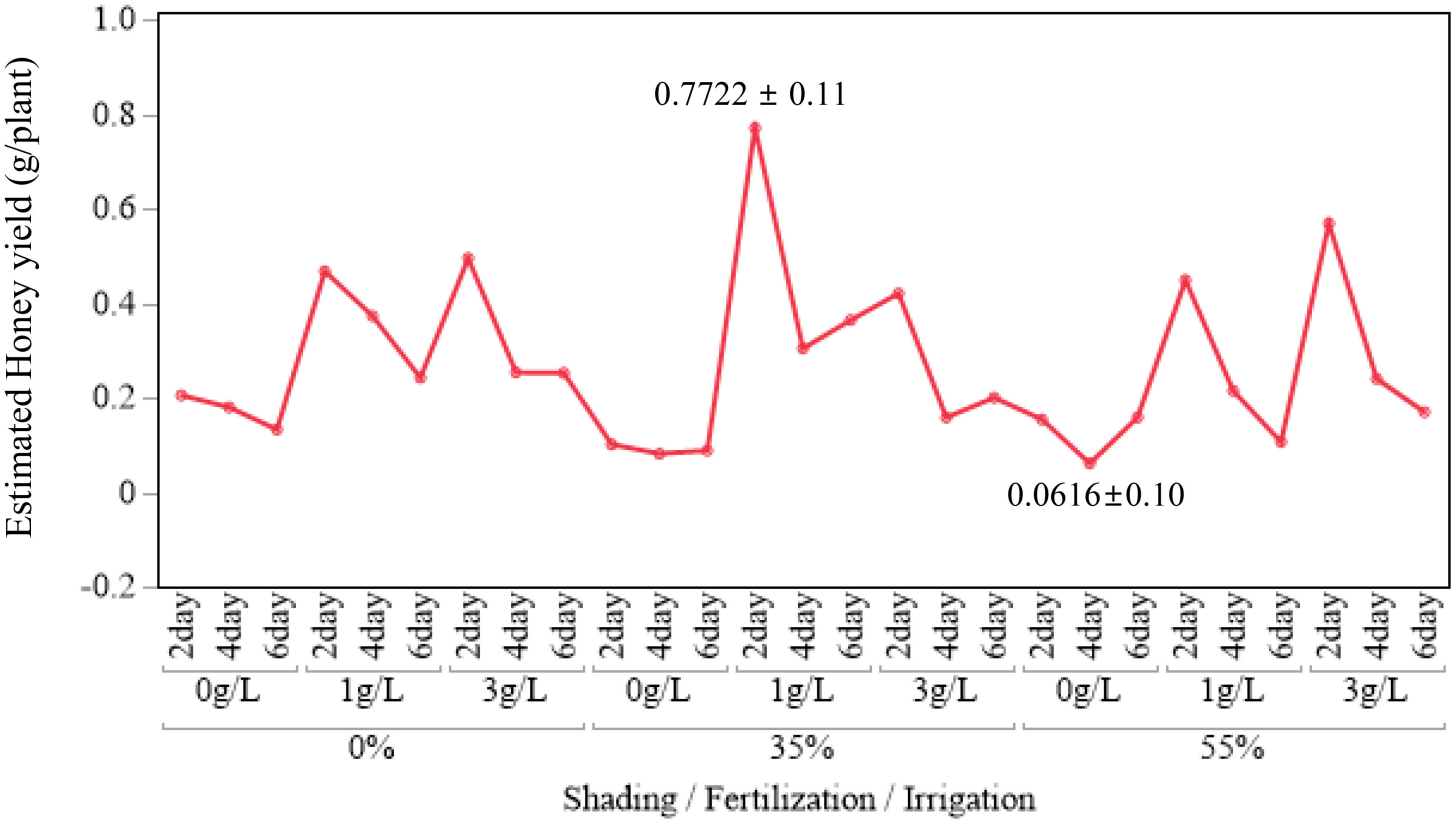
Three-way interaction plot of estimated honey yield (g/plant) across all 27 treatment combinations in *A. rugosa*. Values represent LSM ± SE.

Because none of the shading–fertilization, shading–irrigation, fertilization–irrigation, or three-way combinations were significant for estimated honey yield (all *p* > 0.05), interpretation was restricted to these main-effect patterns. Treatment combinations are therefore reported as descriptive summaries. For example, honey yield per plant reached 0.4809 g at 35 % shading with 1 g/L fertilization and 0.4318 g at 35 % shading with 2-day irrigation. The combination of 1 g/L fertilization with 2-day irrigation yielded 0.5636 g/plant, and the highest observed value across all treatment combinations was 0.7722 g/plant at 35 % shading, 1 g/L fertilization, and 2-day irrigation (**Figures 5**–**7**). These combination-level values illustrate how the main effect trends in fertilization and irrigation are expressed under specific shading conditions, but they are not supported by significant interaction tests.

Post-hoc power analysis for the estimated honey yield indicated high power for detecting fertilization (0.9985) and irrigation effects (0.9966). Power for the fertilization × irrigation term was moderate (0.6636), and power for shading and the remaining interaction terms ranged from 0.1431 to 0.2037 (**Table 7**), consistent with the absence of statistically significant interaction effects.

**Table 7:** Power analysis results for the main effects and interactions affecting honey yield in *A. rugosa*. Values include least significant number (LSN), power estimates, and confidence intervals.

Factor	N	LSN	Power	Observed power	95% CI
Shading	160	664.3	0.1707	0.0500	0.0500, 0.9132
Fertilization	160	41.63	0.9985	0.9969	0.7170, 1.0000
Irrigation	160	44.52	0.9966	0.9932	0.6256, 1.0000
Shading x Fertilization	160	188.8	0.6011	0.3172	0.0500, 0.9992
Shading x Irrigation	160	927.9	0.1431	0.0500	0.0500, 0.9518
Fertilization x Irrigation	160	167.2	0.6636	0.3967	0.0500, 0.9996
Shading x Fertilization x Irrigation	160	694.2	0.2037	0.0500	0.0500, 0.9944

## 4 Discussion

### 4.1 Effects of shading, fertilization, and irrigation on growth, floral traits, and nectar production

This study demonstrated that *A. rugosa* responded consistently to changes in nutrient and water supply, with stronger effects on morphological traits than on nectar traits (**Figure 3**; **Tables 3**-**5**). Plant height, inflorescences per plant, flowers per inflorescence, and total flowers per plant all increased with fertilization and with more frequent irrigation, whereas shading produced smaller and more trait-specific differences (**Figures 3**, **4**; **Tables 4**, **5**). These patterns are consistent with the ANOVA results, in which fertilization and irrigation were significant for all morphological traits, while shading was not significant for plant height or total flowers per plant (**Table 4**).

Shading mainly influenced inflorescences rather than overall plant growth. Longer inflorescences and higher numbers of flowers per inflorescence were observed under 0 % shading compared with 35 %, with partial recovery at 55 % shading (**Supplementary Table 2**). The significant shading × fertilization and shading × irrigation terms for several morphological traits indicate that the magnitude of nutrient and water effects depended on the light environment (**Table 4**). For example, a 2-day irrigation interval increased inflorescence length and flowers per inflorescence at both 0 % and 55 % shading, whereas the same irrigation regime produced smaller gains at 35 % shading (**Supplementary Figure 1**; **Supplementary Table 2**). Similar context-dependent shading effects have been reported for other Lamiaceae herbs such as *Ocimum basilicum* and *Mentha arvensis*, in which partial shade altered reproductive growth and nectar secretion by modifying canopy temperature, leaf energy balance, and vapor pressure deficit (Goldani et al., 2021; Eskandarzade et al., 2023; Bghbani-Arani and Poureisa, 2024).

Recent studies on *A. rugosa* under graded light and fertilizer supply, plants adjust photosynthetic electron transport, redox status, and ATP homeostasis in ways that sustain biomass accumulation and reproductive allocation (Rosli et al., 2024a, Rosli et al., 2024b; Rosli et al., 2025a, Rosli et al., 2025b, Rosli et al., 2025c). Under moderate irradiance, such adjustments are associated with reduced photo-oxidative stress and improved water-use efficiency, conditions that can support the development of additional or longer inflorescences even when plant height changes only slightly. By contrast, stronger shading is expected to limit carbon assimilation and the initiation of floral meristems. In other species, reduced irradiance combined with changes in hormone signaling has been shown to suppress flower formation and yield (Michael Menzel, 2022; Liang et al., 2025; Paponov and Paponov, 2025). In the present experiment, the lower values of total flowers per plant and estimated honey yield at 55 % shading are consistent with a reduced carbon supply to reproductive tissues (**Figures 3**, **5**; **Tables 1**, **6**), although the underlying physiological mechanisms were not measured directly.

The interaction between irrigation and fertilization on plant growth and floral output was also evident in the ANOVA. The fertilization × irrigation term was significant for plant height, inflorescences/plant, and total flowers per plant (**Table 4**). In the case of height, the irrigation interval at which the maximum value occurred shifted with fertilizer level: at 0 g/L, height was greatest at the 4-day interval, at 1 g/L it peaked at 6-days, and at 3 g/L at 2- days (**Supplementary Figure 1**). In contrast, total flowers per plant reached its highest values at the 2-day interval at all three fertilizer levels (**Supplementary Table 1**). Thus, more frequent irrigation was consistently associated with larger floral displays, while additional fertilization increased the number of inflorescences and flowers that could be supported under each watering regime, in line with previous reports that water availability and nutrient supply jointly regulate shoot branching and flower production in aromatic herbs (Carroll et al., 2001; Katsoulas et al., 2006; Marchioni et al., 2020; Gao et al., 2023; Mansinhos et al., 2024).

Nectar traits showed narrower ranges of variation than the morphological traits (**Figures 1**, **4**; **Tables 1**-**3**; **Supplementary Figure 2**). Nectar volume per flower tended to be higher at 0 g/L fertilization, under 0 % shading, and at a 2-day irrigation interval, but the overall differences were small (**Figure 4**; **Supplementary Figure 2**; **Supplementary Table 2**). Free sugar content (µg/µL) remained within a relatively narrow concentration range across all treatments, which aligns with the non-significant omnibus tests (**Tables 3**, **5**). The absence of significant interaction terms for nectar traits suggests that, within the treatment range applied in this study, combined changes in light, nutrient, and water supply had limited effects on nectar volume per flower and free sugar content (De la Barrera and Nobel, 2004; Galetto and Bernardello, 2004) (**Tables 3**, **5**).

### 4.2 Functional relationships between morphological and nectar traits

Across the 27 treatment combinations, morphological traits of *A. rugosa* showed coordinated variation, whereas nectar traits changed much less and exhibited limited covariance with plant growth and flower number (Sections 1.1–1.3; **Figures 2**-**4**; **Tables 1**-**3**). Correlation analysis revealed positive associations among plant height, inflorescences/plant, inflorescence length, flowers per inflorescence, and total flowers per plant (**Figure 2**). In contrast, nectar volume per flower and free sugar content (µg/µL) showed negligible correlations with these structural traits (|r|< 0.07), and the correlation between nectar volume per flower and free sugar content was weakly negative (r = −0.19) (**Figure 2**). Consistent with these patterns, MANOVA and ANOVA indicated strong effects of fertilization and irrigation on morphological traits but no significant effects of shading, fertilization, irrigation, or their interactions on free sugar content and only small, non-systematic differences in nectar volume per flower (Sections 1.3–1.5; **Tables 3**-**5**). Within the range of treatments applied here, these results indicate partial decoupling between structural investment in flowers and the measured nectar properties, in line with evidence that floral morphology and nectar traits can differ in their evolutionary potential (RomeroBravo and Castellanos, 2024) and that nectar production may vary across flowers and plants largely independently of overall plant size or display (Lu et al., 2015; García et al., 2023; Malovrh et al., 2024a).

One possible interpretation is that visual display and nectar secretion in *A. rugosa* are regulated through partially independent control pathways (Hamilton and Lobel, 2008). Studies in related systems have suggested that floral attraction and nectar provisioning can be influenced by different hormonal signals and sink–source relationships within the plant (Lin et al., 2014; Roy et al., 2017; Nicolson, 2022). The present experiment did not measure such mechanisms directly, but the limited covariance between floral display and nectar traits observed here is compatible with a scenario in which nectar production is buffered against moderate variation in plant size and flower number (**Figures 2**-**4**; **Tables 3**-**5**). From a practical perspective, this pattern suggests that fertilization and irrigation regimes that increase inflorescence length and flower number (Sections 1.3–1.5; **Figures 3**, **4**; **Supplementary Figures 1, 2**; **Supplementary Tables 1, 2**) may not necessarily cause large shifts in nectar volume/flower or sugar content/volume (Sections 1.3–1.5; **Figure 4**; **Tables 3**, **5**). Such partial independence offers some flexibility for managing *A. rugosa* as a dual-purpose crop, allowing enhancement of floral display for ornamental or landscape use while maintaining relatively stable nectar properties for pollinators, at least under the environmental conditions tested in this study.

### 4.3 Identifying high-yielding cultivation strategies for honey production

Estimated honey yield per plant responded strongly to fertilization and irrigation regimes, whereas shading had only modest effects (Section 1.6; **Figure 5**; **Table 6**). Honey yield per plant increased from 0.1298 g at 0 g/L to 0.3667 g at 1 g/L and then declined slightly to 0.3076 g at 3 g/L, and a 2-day irrigation interval produced higher yields (0.4046 g/plant) than 4-day (0.2081 g/plant) or 6-day (0.1911 g/plant) intervals (Section 1.6; **Figure 5**; **Table 6**). Shading did not show a significant main effect on honey yield, with similar least squares means at 0 % and 35 % shading and somewhat lower values at 55 % shading (Section 1.6; **Table 6**).

Only the main effects of fertilization and irrigation were significant on estimated honey yield per plant, whereas all interaction terms were not (Section 1.6; **Table 6**). Within this, the highest observed honey yield per plant (0.7722 g/plant, equivalent to 62.1 kg/ha under the assumed planting density) occurred at 35 % shading with 1 g/L fertilization and 2-day irrigation (Sections 1.1, 1.6; **Figures 5**-**7**; **Tables 1**, **6**). The combination of 1 g/L fertilization and 2-day irrigation consistently produced higher yields than either lower fertilization or longer irrigation intervals, with yields of 0.5636 g/plant compared with 0.1298 g/plant at 0 g/L and 0.1911–0.2081 g/plant at 4- and 6-day intervals (Section 1.6; **Figure 5**; **Table 6**). These patterns indicate that, under the controlled conditions of this experiment, moderate fertilization coupled with frequent irrigation is a promising regime for maximizing honey yield from *A. rugosa* (Lerdau and Coley, 2002; Tsirogiannis et al., 2010; Mueller et al., 2020; Kim et al., 2022; Lalevic et al., 2023).

The magnitude of the estimated honey yield under these conditions is comparable to or higher than values reported for several commonly cultivated nectar plants in Korean apicultural systems. For example, estimates for *Perilla frutescens*, *Vicia villosa*, and some *Brassica napus* cultivars often fall below 50 kg/ha under conventional management (Na et al., 2023, Na et al., 2024). Within this context, *A. rugosa* appears to be a competitive candidate for diversifying honey production resources, particularly as an additional late-season nectar source alongside existing species such as *Robinia pseudoacacia*, which has shown declining productivity in some regions due to disease and climatic fluctuations (Kellezi and Kortoci, 2022; Sestras et al., 2025; Yan et al., 2025). However, these comparisons are based on modeled honey yield derived from floral and nectar traits rather than direct hive-level measurements, and field-based validation will be required before extending these conclusions to broader agroecosystem or urban contexts.

### 4.4 Ecological and practical implications for climate-resilient melliferous cropping

Nectar traits of *A. rugosa* showed comparatively low variation across shading, fertilization, and irrigation treatments, whereas morphological and reproductive traits responded strongly to fertilization and irrigation (Sections 1.1, 1.3–1.5; **Figures 1**, **3**, **4**; **Tables 1**-**3**, **5**). Nectar volume per flower and free sugar content (µg/µL) remained within relatively narrow concentration ranges and did not exhibit significant main effects or interactions in the MANOVA and ANOVA models (Sections 1.3–1.5; **Figure 4**; **Tables 3**, **5**). In contrast, many melliferous species show marked changes in nectar volume and sugar concentration under light or water stress (Takkis et al., 2015; Descamps et al., 2021a, Descamps et al., 2021b; Plos et al., 2023; Göttlinger et al., 2025). Within the present pot-based system, this pattern indicates that, over the range of treatments applied here, nectar properties of *A. rugosa* were relatively stable even when plant size and flower number varied substantially (Sections 1.1, 1.3–1.5; **Figures 3**, **4**; **Tables 3**-**5**).

The observed responses of floral display and estimated honey yield suggest practical avenues for the management of *A. rugosa* in production and ornamental systems. Moderate shading (0–35 %) combined with regular irrigation and fertilization at 1–3 g/L increased inflorescence length, flowers per inflorescence, total flowers per plant, and estimated honey yield per plant compared with low nutrient supply or longer irrigation intervals (Sections 1.3, 1.5–1.6; **Figures 3**, **5**; **Tables 4**, **6**). Overall, partial shade netting and scheduled irrigation could be employed in nurseries, gardens, or smallholder systems to enhance inflorescence production and honey yield potential while maintaining relatively consistent nectar properties (Sections 1.3–1.6). The results obtained provide guidance for integrating *A. rugosa* into multifunctional plantings near apiaries or in peri-urban green spaces, where visual appeal and nectar provision for pollinators are both desired (Mueller et al., 2020; Pljevljakusic and Brkic, 2020; Seitz et al., 2022). However, given that the present experiment was conducted in pots under controlled conditions and honey yield was estimated from floral and nectar traits rather than measured at the colony level, further field-based studies will be needed to assess how robust these patterns are under more variable environmental conditions and in complex agroecosystems.

### 4.5 Study limitations and future directions

This study provided a detailed assessment of how controlled abiotic treatments influence the growth and nectar production of *A. rugosa*, but several methodological limitations should be acknowledged. First, the experiment was conducted in pots under greenhouse-like conditions, which may not fully represent the complexity of field environments. Interactions between roots and native soils, spatial microclimatic gradients, and belowground biotic factors such as microbial activity or soil structure were not captured (Philippot et al., 2013). In addition, factors such as localized differences in temperature, humidity, and light within the experimental area, as well as potential nutrient leaching or salt accumulation in pots, may have influenced plant responses in ways that differ from open-field systems. Potential feedbacks between plant traits and pollinator visitation were also excluded, because no active pollinator access was permitted (Herrera, 2000; Descamps et al., 2021a; Plos et al., 2023).

To assess the applicability of these findings to real-world conditions, future studies should be conducted under open-field or semi-natural agroecosystem conditions (Tew et al., 2021; Frigero et al., 2025). It is imperative that such experiments incorporate a greater number of dynamic environmental variables, including, but not limited to, natural variations in temperature, radiation, and soil moisture (Seitz et al., 2022; García et al., 2023). In addition, it is essential that soil physicochemical properties and microbial communities are monitored in order to capture plant–soil feedback (Philippot et al., 2013; Vaudo et al., 2022; Davis et al., 2023). Allowing natural pollinator access would also enable evaluation of how floral traits of *A. rugosa* interact with pollinator behavior and visitation patterns *in situ* (Herrera, 2000; Descamps et al., 2021a; Plos et al., 2023).

Second, honey yield in this study was estimated from floral and nectar traits using standard modeling equations, rather than measured directly through colony-level honey production. While this approach enables standardized comparisons among treatments, it does not capture variation introduced by pollinator foraging behavior, nectar depletion and replenishment cycles, or seasonal shifts in secretion patterns. Future research should therefore include direct observations of pollinator activity, floral visitation rates, and hive-based honey yield to improve ecological realism and validate the modeled estimates (Aksoy et al., 2018; Quinlan et al., 2023). Long-term studies that track plant performance, nectar production, and apiary-level yield across multiple seasons and years would further clarify how stable the identified management regimes are under interannual variation. Taken together, these limitations and proposed extensions provide constructive directions for refining the cultivation and evaluation of *A. rugosa* under practical field conditions and for integrating plant–soil–pollinator interactions into future assessments of its role in multifunctional and climate-adaptive agricultural systems.

## 5 Conclusion

This study demonstrates that integrating the management of light, nutrients, and water can significantly modify the growth, floral display, and honey yield potential of *A. rugosa*, while maintaining the stability of nectar traits across the range of conditions examined. Fertilization and irrigation exhibited robust and consistent effects on plant height, inflorescence traits, and total flower number, while nectar volume and sugar concentration demonstrated minimal variation and did not exhibit significant treatment responses, indicating a partial decoupling between structural investment in flowers and nectar properties. Within this response, moderate fertilization, frequent irrigation, and low to moderate shading were associated with the largest floral displays and the highest modelled honey yields, suggesting management regimes that can enhance both ornamental value and nectar provisioning without evident trade-offs in nectar quality. Collectively, these results identify *A. rugosa* as a promising dual-purpose species for integration into diversified planting schemes, and they provide a quantitative basis for future field-based studies that incorporate soil processes, pollinator behavior, and hive-level honey production to evaluate its performance under more complex agroecosystem conditions.

## Data Availability

The original contributions presented in the study are included in the article/**Supplementary Material**. Further inquiries can be directed to the corresponding author.

## References

[B1] Akplo T. M. Faye A. Obour A. Stewart Z. P. Min D. Prasad P. V. V. (2023). Dual-purpose crops for grain and fodder to improve nutrition security in semi-arid sub-Saharan Africa: A review. Food Energy Secur. 12, e492. doi: 10.1002/fes3.492

[B2] Aksoy A. Ertürk Y. E. Erdogan S. Eyduran E. Tariq M. M. (2018). Estimation of honey production in beekeeping enterprises from eastern part of Turkey through some data mining algorithms. Pak. J. Zool. 50, 2199–2207. doi: 10.17582/journal.pjz/2018.50.6.2199.2207

[B3] Barberis M. Bogo G. Bortolotti L. Flaminio S. Giordano E. Nepi M. . (2022). Nectar Chemistry Changes Over Season in Echium vulgare L. Available online at: https://papers.ssrn.com/sol3/papers.cfm?abstract_id=4220117 (Accessed July 18, 2025).

[B4] Bghbani-Arani A. Poureisa M. (2024). Soil properties and yield of peppermint (Mentha Piperita L.) in response to different nitrogen fertilizers under water-deficit conditions. Commun. Soil Sci. Plant Anal. 55, 1445–1462. doi: 10.1080/00103624.2024.2317852

[B5] Brito Vera G. A. Pérez F. (2024). Floral nectar (FN): drivers of variability, causes, and consequences. Braz. J. Bot. 47, 473–483. doi: 10.1007/s40415-024-01009-8

[B6] Burquez A. Corbet S. A. (1991). Do flowers reabsorb nectar? Funct. Ecol. 5, 369–379. doi: 10.2307/2389808

[B7] Carroll A. B. Pallardy S. G. Galen C. (2001). Drought stress, plant water status, and floral trait expression in fireweed, Epilobium angustifolium (Onagraceae). Am. J. Bot. 88, 438–446. doi: 10.2307/2657108, PMID: 11250821

[B8] Davis J. K. Cohen A. D. Getman-Pickering Z. L. Grab H. L. Hodgden B. Maher R. M. . (2023). Agricultural soil legacy influences multitrophic interactions between crops, their pathogens and pollinators. Proc. R. Soc B. 290, 20231453. doi: 10.1098/rspb.2023.1453, PMID: 38018107 PMC10685131

[B9] De la Barrera E. Nobel P. S. (2004). Nectar: properties, floral aspects, and speculations on origin. Trends Plant Sci. 9, 65–69. doi: 10.1016/j.tplants.2003.12.003, PMID: 15102371

[B10] Descamps C. Quinet M. Jacquemart A.-L. (2021a). climate change–induced stress reduce quantity and alter composition of nectar and pollen from a bee-pollinated species (Borago officinalis, Boraginaceae). Front. Plant Sci. 12. doi: 10.3389/fpls.2021.755843, PMID: 34707633 PMC8542702

[B11] Descamps C. Quinet M. Jacquemart A.-L. (2021b). The effects of drought on plant–pollinator interactions: What to expect? Environ. Exp. Bot. 182, 104297. doi: 10.1016/j.envexpbot.2020.104297

[B12] Díaz-Calafat J. Felton A. Öckinger E. De Frenne P. Cousins S. A. Hedwall P.-O. (2025). The effects of climate change on boreal plant-pollinator interactions are largely neglected by science. Basic. Appl. Ecol. 84, 1–13. doi: 10.1016/j.baae.2025.01.014

[B13] Eskandarzade P. Zare Mehrjerdi M. Didaran F. Gruda N. S. Aliniaeifard S. (2023). Shading level and harvest time affect the photosynthetic and physiological properties of basil varieties. Agronomy 13, 2478. doi: 10.3390/agronomy13102478

[B14] Fernandes K. E. Stanfield B. Frost E. A. Shanahan E. R. Susantio D. Dong A. Z. . (2023). Low levels of hive stress are associated with decreased honey activity and changes to the gut microbiome of resident honeybees. Microbiol. Spectr. 11, e00742–e00723. doi: 10.1128/spectrum.00742-23, PMID: 37289060 PMC10434159

[B15] Frigero M. L. P. Boaro C. S. Galetto L. Tunes P. Guimarães E. (2025). Extreme events induced by climate change alter nectar offer to pollinators in cross pollination-dependent crops. Sci. Rep. 15, 10852. doi: 10.1038/s41598-025-94565-2, PMID: 40157983 PMC11954887

[B16] Fründ J. Linsenmair K. E. Blüthgen N. (2010). Pollinator diversity and specialization in relation to flower diversity. Oikos 119, 1581–1590. doi: 10.1111/j.1600-0706.2010.18450.x

[B17] Galetto L. Bernardello G. (2004). Floral nectaries, nectar production dynamics and chemical composition in six Ipomoea species (Convolvulaceae) in relation to pollinators. Ann. Bot. 94, 269–280. doi: 10.1093/aob/mch137, PMID: 15229123 PMC4242162

[B18] Gao R. Hu B. Yuan Y. He M. Wang R. Lou Y. . (2023). Nitrogen addition affects floral and vegetative traits, reproduction, and pollinator performance in Capsicum annuum L. Ann. Bot. 132, 1131–1144. doi: 10.1093/aob/mcad121, PMID: 37638856 PMC10809046

[B19] García Y. Dow B. S. Parachnowitsch A. L. (2023). Water deficit changes patterns of selection on floral signals and nectar rewards in the common morning glory. AoB. Plants 15, plad061. doi: 10.1093/aobpla/plad061, PMID: 37899982 PMC10601024

[B20] Goldani M. Bannayan M. Yaghoubi F. (2021). Crop water productivity and yield response of two greenhouse basil (Ocimum basilicum L.) cultivars to deficit irrigation. Water Supply. 21, 3735–3751. doi: 10.2166/ws.2021.134

[B21] Göttlinger T. Naegel D. Dick J. E. Lohaus G. (2025). Influence of drought stress on the metabolite and ion composition in nectar and nectaries of different day- and night-flowering Nicotiana species. Plant Biol. J. doi: 10.1111/plb.70000, PMID: 39963798 PMC13089589

[B22] Hamilton J. G. Lobel M. (2008). Types, patterns, and predictors of coping with stress during pregnancy: examination of the revised prenatal coping inventory in a diverse sample. J. Psychosom. Obstet. Gynaecol. 29, 97–104. doi: 10.1080/01674820701690624, PMID: 18484440

[B23] Herrera C. M. (2000). Flower-to-seedling consequences of different pollination regimes in an insect-pollinated shrub. Ecology 81, 15–29. doi: 10.1890/0012-9658(2000)081[0015:FTSCOD]2.0.CO;2

[B24] Hicks D. M. Ouvrard P. Baldock K. C. Baude M. Goddard M. A. Kunin W. E. . (2016). Food for pollinators: quantifying the nectar and pollen resources of urban flower meadows. PloS One 11, e0158117. doi: 10.1371/journal.pone.0158117, PMID: 27341588 PMC4920406

[B25] Hong M. J. Kim J. H. Kim H. Y. Kim M. J. Kim S. M. (2020). Chemical composition and biological activity of essential oil of Agastache rugosa (Fisch. & CA Mey.) O. Kuntze. Kor. J. Med. Crop Sci. 28, 95–110. doi: 10.7783/KJMCS.2020.28.2.95

[B26] Horga V.-A. Suciu D.-L. Hulujan I.-B. Costin A. D. Ciontea S. Ș Vârban D. . (2024). Therapeutic properties and use for medicinal purposes of agastache species. Hop. Med. Plants 32, 22–34. doi: 10.15835/hpm.v32i1-2.15017

[B27] Katsoulas N. Kittas C. Dimokas G. Lykas C. (2006). Effect of irrigation frequency on rose flower production and quality. Biosyst. Eng. 93, 237–244. doi: 10.1016/j.biosystemseng.2005.11.006

[B28] Kellezi M. K. Kortoci Y. (2022). Comparison of growth rate of black locust (Robinia pseudoacacia L.) on productive and marginal cultivated lands for sustainable agroforestry systems. Ecol. Eng. Environ. Technol. 23, 206–212. doi: 10.12912/27197050/145612

[B29] Kim S. Noh S. Park J. (2022). Increased antioxidants of Agastache rugosa by the night interruption time. J. Bio-Env. Con. 31, 319–324. doi: 10.12791/ksbec.2022.31.4.319

[B30] Lalević D. Ilić Z. S. Stanojević L. Milenković L. Šunić L. Kovač R. . (2023). Shade-induced effects on essential oil yield, chemical profiling, and biological activity in some Lamiaceae plants cultivated in Serbia. Horticulturae 9, 84. doi: 10.3390/horticulturae9010084

[B31] Lerdau M. Coley P. D. (2002). Benefits of the carbon-nutrient balance hypothesis. Oikos 98, 534–536. doi: 10.1034/j.1600-0706.2002.980318.x

[B32] Liang X.-G. Chen H.-M. Pan Y.-Q. Wang Z.-W. Huang C. Chen Z.-Y. . (2025). Yield more in the shadow: Mitigating shading-induced yield penalty of maize via optimizing source-sink carbon partitioning. Eur. J. Agron. 162, 127421. doi: 10.1016/j.eja.2024.127421

[B33] Lin I. W. Sosso D. Chen L.-Q. Gase K. Kim S.-G. Kessler D. . (2014). Nectar secretion requires sucrose phosphate synthases and the sugar transporter SWEET9. Nature 508, 546–549. doi: 10.1038/nature13082, PMID: 24670640

[B34] Lu N. N. Li X. H. Li L. Zhao Z. G. (2015). Variation of nectar production in relation to plant characteristics in protandrous Aconitum gymnandrum. J. Plant Ecol. 8, 122–129. doi: 10.1093/jpe/rtv020

[B35] Malovrh K. Bavcon J. Križman M. Ravnjak B. (2024a). Effect of abiotic factors on nectar quality and secretion of two early spring species, Galanthus nivalis L. and Helleborus Niger L. Diversity 16, 469. doi: 10.3390/d16080469

[B36] Malovrh K. Ravnjak B. Bavcon J. Križman M. (2024b). Nectar production and three main sugars in nectar of Salvia pratensis and Salvia glutinosa in correlation with abiotic factors. Agriculture 14, 668. doi: 10.3390/agriculture14050668

[B37] Mansinhos I. Gonçalves S. Romano A. (2024). How climate change-related abiotic factors affect the production of industrial valuable compounds in Lamiaceae plant species: a review. Front. Plant Sci. 15. doi: 10.3389/fpls.2024.1370810, PMID: 39049861 PMC11266143

[B38] Marchioni I. Najar B. Ruffoni B. Copetta A. Pistelli L. Pistelli L. (2020). Bioactive compounds and aroma profile of some Lamiaceae edible flowers. Plants 9, 691. doi: 10.3390/plants9060691, PMID: 32481758 PMC7356345

[B39] McGrath S. R. Behrendt R. Friend M. A. Moore A. D. (2021). Utilising dual-purpose crops effectively to increase profit and manage risk in meat production systems. Anim. Prod. Sci. 61, 1049–1061. doi: 10.1071/AN20495

[B40] Michael Menzel C. (2022). A review of productivity in strawberry: do the plants need larger canopies, more flowers, or higher CO2 assimilation for higher yields? J. Hortic. Sci. Biotechnol. 97, 674–696. doi: 10.1080/14620316.2022.2077240

[B41] Mueller A. L. Berger C. A. Schittenhelm S. Stever-Schoo B. Dauber J. (2020). Water availability affects nectar sugar production and insect visitation of the cup plant Silphium perfoliatum L. (Asteraceae). J. Agron. Crop Sci. 206, 529–537. doi: 10.1111/jac.12406

[B42] Na S. J. Choi H. M. Park J. M. Choi Y. I. Kim Y. K. (2023). Evaluation of the honey plant potential of five cultivated crops. J. Apic. 38, 267–274. doi: 10.17519/apiculture.2023.09.38.3.267

[B43] Na S.-J. Kim Y.-K. Park J.-M. (2024). Nectar characteristics and honey production potential of five rapeseed cultivars and two wildflower species in South Korea. Plants 13, 419. doi: 10.3390/plants13030419, PMID: 38337952 PMC10856812

[B44] Na S.-J. Park J.-M. Kwon H.-Y. Kim Y.-K. (2025). Assessment of floral nectar and amino acid yield in eight landscape trees for enhanced pollinator food resources in urban forests. Plants 14, 1924. doi: 10.3390/plants14131924, PMID: 40647933 PMC12252262

[B45] Nam H.-H. Kim J. S. Lee J. Seo Y. H. Kim H. S. Ryu S. M. . (2020). Pharmacological effects of Agastache rugosa against gastritis using a network pharmacology approach. Biomolecules 10, E1298. doi: 10.3390/biom10091298, PMID: 32916904 PMC7565599

[B46] Nechita M.-A. Toiu A. Benedec D. Hanganu D. Ielciu I. Oniga O. . (2023). Agastache Species: A comprehensive review on phytochemical composition and therapeutic properties. Plants 12, 2937. doi: 10.3390/plants12162937, PMID: 37631149 PMC10459224

[B47] Nepi M. Guarnieri M. Pacini E. (2001). Nectar secretion, reabsorption, and sugar composition in male and female flowers of Cucurbita pepo. Int. J. Plant Sci. 162, 353–358. doi: 10.1086/319581

[B48] Nicolson S. W. (2022). Sweet solutions: nectar chemistry and quality. Phil. Trans. R. Soc B. 377, 20210163. doi: 10.1098/rstb.2021.0163, PMID: 35491604 PMC9058545

[B49] Oliveira W. Cruz-Neto O. Silva J. L. S. Tabarelli M. Peres C. A. Lopes A. V. (2025). Climate change will lead to local extinctions and mismatched range contractions disrupting bee-dependent crop pollination. Front. Bee. Sci. 3. doi: 10.3389/frbee.2025.1510451

[B50] Olsen S. L. Evju M. Åström J. Løkken J. O. Dahle S. Andresen J. L. . (2022). Climate influence on plant–pollinator interactions in the keystone species Vaccinium myrtillus. Ecol. Evol. 12, e8910. doi: 10.1002/ece3.8910, PMID: 35619731 PMC9126989

[B51] Pamminger T. Becker R. Himmelreich S. Schneider C. W. Bergtold M. (2019). The nectar report: quantitative review of nectar sugar concentrations offered by bee visited flowers in agricultural and non-agricultural landscapes. PeerJ 7, e6329. doi: 10.7717/peerj.6329, PMID: 30834180 PMC6397631

[B52] Paponov I. A. Paponov M. (2025). Supplemental lighting in controlled environment agriculture: Enhancing photosynthesis, growth, and sink activity. CABI. Rev. 20, 0008. doi: 10.1079/cabireviews.2025.0008

[B53] Park J.-M. Na S.-J. Kwon H.-Y. Kim Y.-K. (2025). Evaluation of nectar characteristics and potential honey production of Robinia pseudoacacia. J. Kor. For. Soc. 114, 84–93. doi: 10.14578/jkfs.2025.114.1.84

[B54] Petanidou T. (2003). Introducing plants for bee-keeping at any cost? – Assessment of Phacelia tanacetifolia as nectar source plant under xeric Mediterranean conditions. Plant Syst. Evol. 238, 155–168. doi: 10.1007/s00606-002-0278-x

[B55] Petanidou T. (2007). “ Ecological and evolutionary aspects of floral nectars in Mediterranean habitats,” in Nectaries and Nectar ( Springer Netherlands, Dordrecht), 343–375. doi: 10.1007/978-1-4020-5937-7_8

[B56] Philippot L. Raaijmakers J. M. Lemanceau P. van der Putten W. H. (2013). Going back to the roots: the microbial ecology of the rhizosphere. Nat. Rev. Microbiol. 11, 789–799. doi: 10.1038/nrmicro3109, PMID: 24056930

[B57] Pljevljakušić D. Brkić S. (2020). Cultivation cost-benefit analysis of some important medicinal plants in Serbia. Lek. Sirov. 40, 13–21. doi: 10.5937/leksir2040013P

[B58] Plos C. Stelbrink N. Römermann C. Knight T. M. Hensen I. (2023). Abiotic conditions affect nectar properties and flower visitation in four herbaceous plant species. Flora 303, 152279. doi: 10.1016/j.flora.2023.152279

[B59] Quinlan G. M. Miller D. A. Grozinger C. M. (2023). Examining spatial and temporal drivers of pollinator nutritional resources: evidence from five decades of honeybee colony productivity data. Environ. Res. Lett. 18, 114018. doi: 10.1088/1748-9326/acff0c

[B60] RomeroBravo A. Castellanos M. C. (2024). Nectar and floral morphology differ in evolutionary potential in novel pollination environments. New Phytol. 243, 753–764. doi: 10.1111/nph.19780, PMID: 38714871

[B61] Rosli K. A. Misran A. Saiful Yazan L. Megat Wahab P. E. (2025a). High-light and nutrient interactions drive carbohydrate and proton pump dynamics in Agastache rugosa (Fisch. & C.A.Mey.) Kuntze. Plant Physiol. Biochem. 219, 109374. doi: 10.1016/j.plaphy.2024.109374, PMID: 39709665

[B62] Rosli K. A. Misran A. Yazan L. S. Wahab P. E. M. (2024a). Integrative physiological plasticity of Agastache rugosa (Fisch. & C.A. Mey.) Kuntze reveals complex adaptation to light and nutrient gradients. bioRxiv. 10.01.616001. doi: 10.1101/2024.10.01.616001

[B63] Rosli K. A. Misran A. Yazan L. S. Wahab P. E. M. (2024b). Light–NPK synergy increases biomass, photosynthetic pigment and nitrogen content in Agastache rugosa (Fisch. & C.A. Mey.) Kuntze. J. Trop. Plant Physiol. 16, 26–26. doi: 10.56999/jtpp.2024.16.2.3

[B64] Rosli K. A. Misran A. Yazan L. S. Wahab P. E. M. (2025b). Light and nutrient cues elicit metabolic reprogramming by targeting carbon fixation, redox balance, and ATP homeostasis in Agastache rugosa. Planta 261, 133. doi: 10.1007/s00425-025-0133-x, PMID: 40347293

[B65] Rosli K. A. Misran A. Yazan L. S. Wahab P. E. M. (2025c). Multivariate analysis reveals physiological trade-offs and synergies under light and nutrient gradients in the herbaceous species Agastache rugosa. Funct. Plant Biol. 52, FP24323. doi: 10.1071/FP23328, PMID: 40479518

[B66] Roy R. Schmitt A. J. Thomas J. B. Carter C. J. (2017). Review: Nectar biology: from molecules to ecosystems. Plant Sci. 262, 148–164. doi: 10.1016/j.plantsci.2017.04.012, PMID: 28716410

[B67] Rural Development Administration (RDA) (2018). “ Baechohyang (Agastache rugosa) – Basic information and cultivation methods,” in New Resource Crops, Chapter 5: Baechohyang ( Nongsaro, Rural Development Administration, Jeonju, Korea), 74.

[B68] Seitz B. Buchholz S. Kowarik I. Herrmann J. Neuerburg L. Wendler J. . (2022). Land sharing between cultivated and wild plants: urban gardens as hotspots for plant diversity in cities. Urban. Ecosyst. 25, 927–939. doi: 10.1007/s11252-021-01198-0

[B69] Sestras A. F. Sălăgean T. Roman A. M. Morar I. M. Dan C. Truta A. M. . (2025). Growth and resistance to mechanical stress in the young phase of black locust (Robinia pseudoacacia L.) trees based on geographical provenances. J. Environ. Manag. 384, 125465. doi: 10.1016/j.jenvman.2025.125465, PMID: 40300542

[B70] Takkis K. Tscheulin T. Tsalkatis P. Petanidou T. (2015). Climate change reduces nectar secretion in two common Mediterranean plants. AoB. Plants 7, plv111. doi: 10.1093/aobpla/plv111, PMID: 26374517 PMC4614813

[B71] Tew N. E. Memmott J. Vaughan I. P. Bird S. Stone G. N. Potts S. G. . (2021). Quantifying nectar production by flowering plants in urban and rural landscapes. J. Ecol. 109, 1747–1757. doi: 10.1111/1365-2745.13598

[B72] Tsirogiannis I. Katsoulas N. Kittas C. (2010). Effect of irrigation scheduling on gerbera flower yield and quality. HortScience 45, 265–270. doi: 10.21273/HORTSCI.45.2.265

[B73] Vannette R. L. (2020). The Floral Microbiome: Plant, pollinator, and microbial perspectives. Annu. Rev. Ecol. Evol. Syst. 51, 363–386. doi: 10.1146/annurev-ecolsys-011720-013401

[B74] Vaudo A. D. Erickson E. Patch H. M. Grozinger C. M. Mu J. (2022). Impacts of soil nutrition on floral traits, pollinator attraction, and fitness in cucumbers (Cucumis sativus L.). Sci. Rep. 12, 21802. doi: 10.1038/s41598-022-26164-4, PMID: 36526706 PMC9758155

[B75] Wang Y. Zhao R. Zhou X. Zhang X. Zhao G. Zhang F. (2023). Prediction of potential distribution areas and priority protected areas of Agastache rugosa based on Maxent model and Marxan model. Front. Plant Sci. 14. doi: 10.3389/fpls.2023.1200796, PMID: 37554556 PMC10405825

[B76] Xing Z. Bi G. Li T. Zhang Q. Knight P. R. (2023). Nitrogen fertilization improves growth and bioactive compound content for Salvia miltiorrhiza Bunge. Horticulturae 9, 254. doi: 10.3390/horticulturae9020254

[B77] Yan X. Zhang Z. Wu X. Huang M. (2025). Radial growth-climate correlations and resilience of Robinia pseudoacacia plantations to drought on the Chinese Loess Plateau. Front. For. Glob. Change 8. doi: 10.3389/ffgc.2025.1608397

[B78] Yousefzadeh K. Houshmand S. Shiran B. Mousavi-Fard S. Zeinali H. Nikoloudakis N. . (2022). Joint effects of developmental stage and water deficit on essential oil traits (content, yield, composition) and related gene expression: A case study in two Thymus species. Agronomy 12, 1008. doi: 10.3390/agronomy12051008

[B79] Zhang G. St Clair A. L. Dolezal A. G. Toth A. L. O’Neal M. E. (2022). Can native plants mitigate climate-related forage dearth for honeybees (Hymenoptera: Apidae)? J. Econ. Entomol. 115, 1–9. doi: 10.1093/jee/toab202, PMID: 34850022 PMC8827321

[B80] Zielińska S. Matkowski A. (2014). Phytochemistry and bioactivity of aromatic and medicinal plants from the genus Agastache (Lamiaceae). Phytochem. Rev. 13, 391–416. doi: 10.1007/s11101-014-9349-1, PMID: 24899872 PMC4032471

