# Supplementary material for: Effects of shading, fertilization, and irrigation on floral display and honey yield in Agastache rugosa in controlled pot culture
Source: Front Plant Sci. 2025 Dec 16;16:1679318. doi: 10.3389/fpls.2025.1679318 (PMC12750111; doi:10.3389/fpls.2025.1679318)
Supplement: Supplementary file 1 [file DataSheet1.docx]

Supplementary Material

## Supplementary Figures


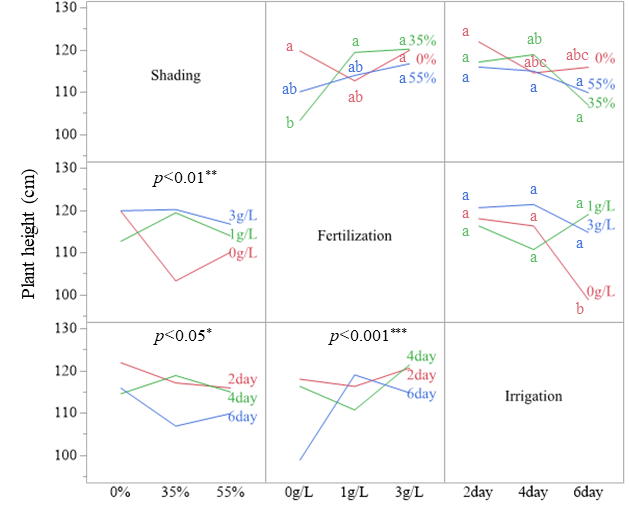

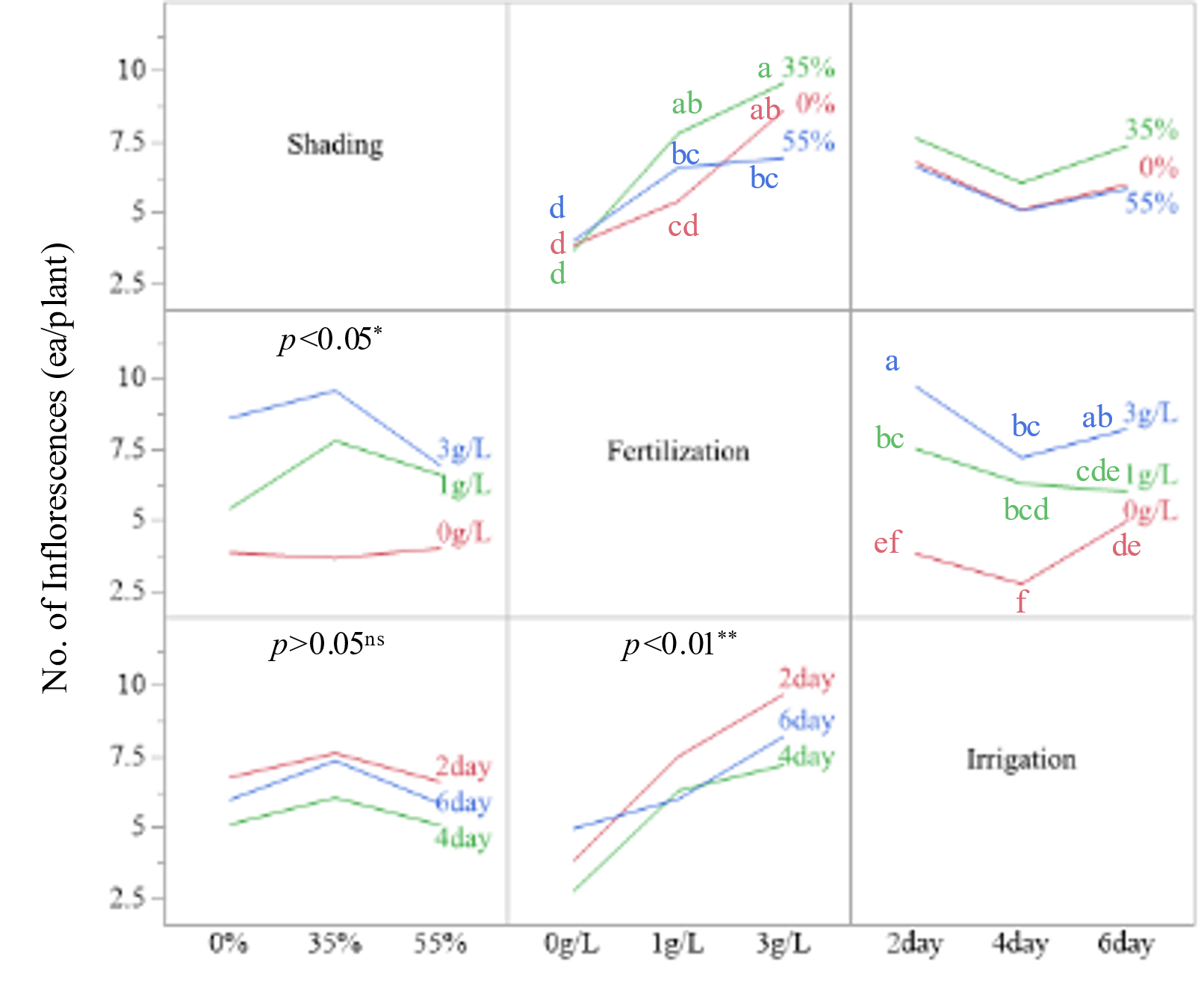


(A)

(B)


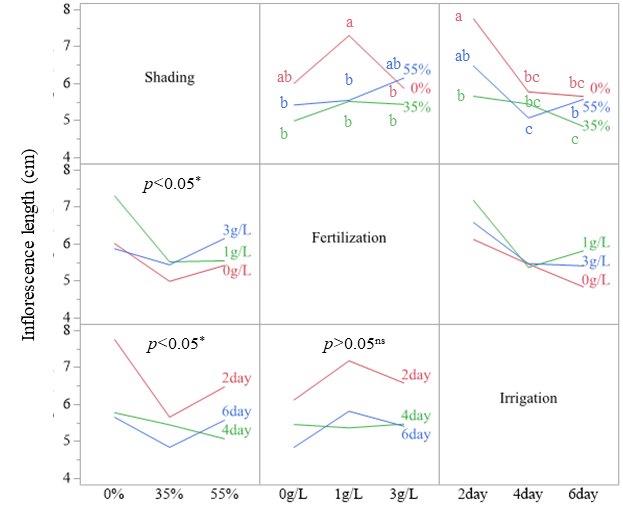

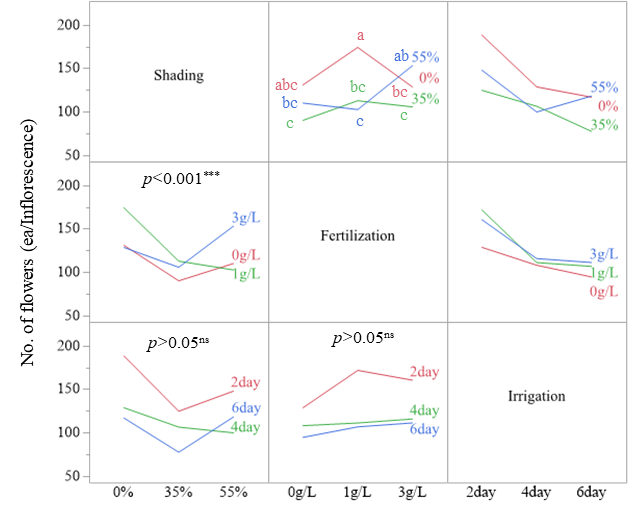

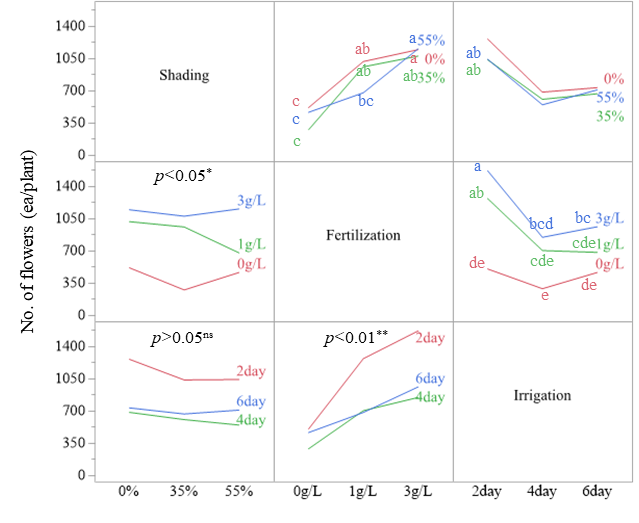


(C)

(D)

(E)

Supplementary Figure 1. Two‑way interaction plots for (A) plant height (cm), (B) inflorescences per plant, (C) inflorescence length (cm), (D) flowers per inflorescence, and (E) total flowers per plant of *A. rugosa* across combinations of shading, fertilization, and irrigation. Lines connect least squares means (LSM) across factor levels. Where interactions were significant (*p* < 0.05), different letters indicate Tukey‑adjusted groupings.


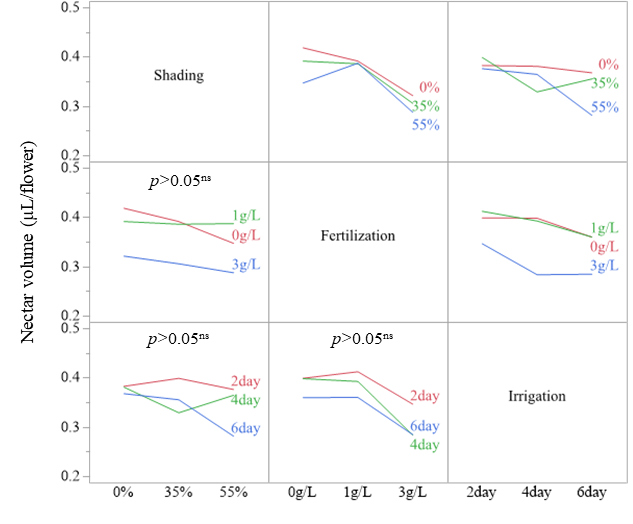


(B)

(A)


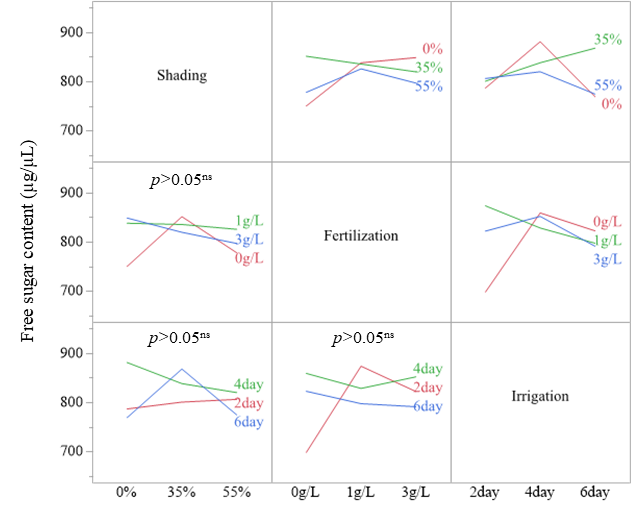


Supplementary Figure 2. Two‑way interaction plots for (A) nectar volume per flower and (B) free sugar content (µg/µL) of *A. rugosa* across combinations of shading, fertilization, and irrigation. Lines connect least squares means (LSM) across factor levels.

## Supplementary Table

Supplementary Table 1. Least squares means (LSM) from the MANOVA for morphological and nectar traits of *A. rugosa* across 27 treatment combinations. Treatments represent the factorial combinations of three shading levels (S; 0, 35, 55%), three fertilization levels (F; 0, 1, 3 g/L), and three irrigation intervals (I; 2-, 4-, 6-days).

| **Treatment** | **level** | **Plant Height (cm)** | **No. of Inflorescences (ea/plant)** | **Inflorescence Length (cm)** | **No. of Flowers (ea/Inflorescence)** | **No. of Flowers (ea/plant)** | **Nectar Volume (µL/flower)** | **Free Sugar Content (µg/µL)** |
| --- | --- | --- | --- | --- | --- | --- | --- | --- |
| S | 0% | 116.97 | 5.93 | 6.05 | 142.21 | 911.13 | 0.37 | 814.24 |
|  | 35% | 114.34 | 7.69 | 5.33 | 102.37 | 864.10 | 0.36 | 835.20 |
|  | 55% | 112.98 | 5.87 | 5.53 | 118.78 | 768.86 | 0.34 | 799.94 |
| F | 0g/L | 110.50 | 3.69 | 5.24 | 107.02 | 397.93 | 0.38 | 795.23 |
|  | 1g/L | 113.35 | 7.30 | 5.84 | 127.33 | 984.37 | 0.39 | 832.75 |
|  | 3g/L | 120.44 | 8.50 | 5.82 | 129.00 | 1,161.80 | 0.30 | 821.40 |
| I | 2day | 117.02 | 7.55 | 6.45 | 151.28 | 1,214.41 | 0.39 | 797.57 |
|  | 4day | 116.53 | 5.46 | 5.48 | 113.06 | 644.49 | 0.36 | 848.35 |
|  | 6day | 110.74 | 6.48 | 4.98 | 99.02 | 685.19 | 0.33 | 803.46 |
| S×F | 0%,0g/L | 120.30 | 3.93 | 5.87 | 131.14 | 525.26 | 0.41 | 756.87 |
|  | 0%,1g/L | 109.82 | 5.92 | 6.36 | 156.83 | 1,023.97 | 0.39 | 837.63 |
|  | 0%,3g/L | 120.78 | 7.94 | 5.92 | 138.65 | 1,184.17 | 0.32 | 848.22 |
|  | 35%,0g/L | 103.75 | 3.32 | 4.65 | 83.77 | 239.60 | 0.39 | 851.04 |
|  | 35%,1g/L | 118.62 | 9.41 | 5.98 | 128.20 | 1,281.44 | 0.39 | 835.21 |
|  | 35%,3g/L | 120.67 | 10.33 | 5.36 | 95.14 | 1,071.26 | 0.31 | 819.34 |
|  | 55%,0g/L | 107.44 | 3.83 | 5.21 | 106.16 | 428.93 | 0.35 | 777.79 |
|  | 55%,1g/L | 111.61 | 6.56 | 5.18 | 96.95 | 647.69 | 0.39 | 825.40 |
|  | 55%,3g/L | 119.89 | 7.22 | 6.19 | 153.22 | 1,229.96 | 0.29 | 796.64 |
| S×I | 0%,2day | 119.88 | 6.37 | 7.24 | 179.43 | 1,275.34 | 0.38 | 786.46 |
|  | 0%,4day | 116.24 | 5.49 | 5.74 | 130.54 | 741.17 | 0.37 | 887.55 |
|  | 0%,6day | 114.78 | 5.94 | 5.17 | 116.65 | 716.89 | 0.37 | 768.71 |
|  | 35%,2day | 116.86 | 9.18 | 5.62 | 125.56 | 1,265.90 | 0.40 | 800.36 |
|  | 35%,4day | 119.50 | 6.00 | 5.67 | 109.12 | 656.19 | 0.33 | 837.88 |
|  | 35%,6day | 106.68 | 7.88 | 4.70 | 72.43 | 670.21 | 0.36 | 867.35 |
|  | 55%,2day | 114.33 | 7.11 | 6.48 | 148.83 | 1,102.00 | 0.38 | 805.89 |
|  | 55%,4day | 113.83 | 4.89 | 5.03 | 99.51 | 536.11 | 0.36 | 819.61 |
|  | 55%,6day | 110.78 | 5.61 | 5.06 | 107.99 | 668.46 | 0.28 | 774.33 |
| F×I | 0g/L,2day | 116.78 | 3.83 | 5.85 | 125.30 | 488.70 | 0.40 | 697.94 |
|  | 0g/L,4day | 118.41 | 2.88 | 5.58 | 103.99 | 298.97 | 0.39 | 865.48 |
|  | 0g/L,6day | 96.30 | 4.37 | 4.30 | 91.79 | 406.11 | 0.36 | 822.28 |
|  | 1g/L,2day | 109.89 | 8.33 | 6.31 | 157.75 | 1,384.08 | 0.41 | 873.07 |
|  | 1g/L,4day | 110.56 | 6.56 | 5.66 | 121.56 | 785.28 | 0.39 | 828.00 |
|  | 1g/L,6day | 119.61 | 7.00 | 5.55 | 102.68 | 783.74 | 0.36 | 797.18 |
|  | 3g/L,2day | 124.40 | 10.49 | 7.19 | 170.78 | 1,770.46 | 0.35 | 821.69 |
|  | 3g/L,4day | 120.61 | 6.94 | 5.20 | 113.63 | 849.22 | 0.28 | 851.56 |
|  | 3g/L,6day | 116.32 | 8.07 | 5.08 | 102.61 | 865.71 | 0.28 | 790.94 |
| S×F×I | 0%,0g/L,2day | 120.00 | 4.17 | 7.17 | 152.64 | 675.56 | 0.42 | 625.66 |
|  | 0%,0g/L,4day | 129.40 | 3.80 | 5.83 | 122.40 | 453.73 | 0.41 | 917.07 |
|  | 0%,0g/L,6day | 111.50 | 3.83 | 4.60 | 118.39 | 446.50 | 0.40 | 727.88 |
|  | 0%,1g/L,2day | 113.80 | 5.60 | 7.03 | 196.20 | 1,351.87 | 0.42 | 880.80 |
|  | 0%,1g/L,4day | 101.17 | 5.67 | 6.23 | 159.11 | 937.56 | 0.39 | 886.03 |
|  | 0%,1g/L,6day | 114.50 | 6.50 | 5.82 | 115.17 | 782.50 | 0.37 | 746.07 |
|  | 0%,3g/L,2day | 125.83 | 9.33 | 7.53 | 189.44 | 1,798.61 | 0.31 | 852.92 |
|  | 0%,3g/L,4day | 118.17 | 7.00 | 5.16 | 110.11 | 832.22 | 0.32 | 859.55 |
|  | 0%,3g/L,6day | 118.33 | 7.50 | 5.08 | 116.39 | 921.67 | 0.33 | 832.18 |
|  | 35%,0g/L,2day | 115.17 | 3.33 | 4.53 | 90.36 | 258.50 | 0.42 | 802.23 |
|  | 35%,0g/L,4day | 112.50 | 2.33 | 5.75 | 106.31 | 220.50 | 0.38 | 909.08 |
|  | 35%,0g/L,6day | 83.57 | 4.29 | 3.68 | 54.64 | 239.79 | 0.37 | 841.80 |
|  | 35%,1g/L,2day | 116.20 | 10.40 | 6.34 | 161.67 | 1,742.87 | 0.39 | 888.05 |
|  | 35%,1g/L,4day | 117.00 | 7.67 | 5.81 | 116.56 | 891.67 | 0.37 | 808.93 |
|  | 35%,1g/L,6day | 122.67 | 10.17 | 5.81 | 106.39 | 1,209.78 | 0.40 | 808.65 |
|  | 35%,3g/L,2day | 119.20 | 13.80 | 6.00 | 124.67 | 1,796.33 | 0.39 | 710.80 |
|  | 35%,3g/L,4day | 129.00 | 8.00 | 5.46 | 104.50 | 856.39 | 0.23 | 795.62 |
|  | 35%,3g/L,6day | 113.80 | 9.20 | 4.61 | 56.27 | 561.07 | 0.30 | 951.60 |
|  | 55%,0g/L,2day | 115.17 | 4.00 | 5.86 | 132.89 | 532.06 | 0.36 | 665.93 |
|  | 55%,0g/L,4day | 113.33 | 2.50 | 5.15 | 83.25 | 222.67 | 0.37 | 770.30 |
|  | 55%,0g/L,6day | 93.83 | 5.00 | 4.62 | 102.33 | 532.06 | 0.31 | 897.16 |
|  | 55%,1g/L,2day | 99.67 | 9.00 | 5.56 | 115.39 | 1,057.50 | 0.43 | 850.37 |
|  | 55%,1g/L,4day | 113.50 | 6.33 | 4.96 | 89.00 | 526.61 | 0.42 | 789.04 |
|  | 55%,1g/L,6day | 121.67 | 4.33 | 5.01 | 86.47 | 358.94 | 0.31 | 836.80 |
|  | 55%,3g/L,2day | 128.17 | 8.33 | 8.03 | 198.22 | 1,716.44 | 0.34 | 901.37 |
|  | 55%,3g/L,4day | 114.67 | 5.83 | 4.99 | 126.28 | 859.06 | 0.29 | 899.50 |
|  | 55%,3g/L,6day | 116.83 | 7.50 | 5.56 | 135.17 | 1,114.39 | 0.22 | 589.04 |

Supplementary Table 2. Least squares means (LSM) from the three‑way interaction for plant height (cm), inflorescences per plant, inflorescence length (cm), flowers per inflorescence, and total flowers per plant, and for nectar volume per flower and free sugar content (µg/µL) of *A. rugosa* across combinations of shading, fertilization, and irrigation. When interactions were significant, simple‑effect LSM were compared using Tukey adjustment and different letters indicate significant differences; when interactions were not significant, level‑wise LSM are reported without letter groupings for the three shading levels (S; 0, 35, 55%), three fertilization levels (F; 0, 1, 3 g/L), and three irrigation intervals (I; 2-, 4-, 6-days).

| Trait | Treatment  (S x F x I) | Tukey group | | | | | | | LSM | SE |
| --- | --- | --- | --- | --- | --- | --- | --- | --- | --- | --- |
| Plant height | 35%,3g/L,4day |  |  |  |  |  |  |  | 130.1 | 4.217 |
|  | 0%,0g/L,4day |  |  |  |  |  |  |  | 125.1 | 4.619 |
|  | 55%,3g/L,2day |  |  |  |  |  |  |  | 122.9 | 4.619 |
|  | 0%,1g/L,2day |  |  |  |  |  |  |  | 122.7 | 4.217 |
|  | 0%,0g/L,2day |  |  |  |  |  |  |  | 121.7 | 4.217 |
|  | 35%,1g/L,6day |  |  |  |  |  |  |  | 121.4 | 4.404 |
|  | 55%,1g/L,6day |  |  |  |  |  |  |  | 121.3 | 4.217 |
|  | 0%,3g/L,2day |  |  |  |  |  |  |  | 121.2 | 4.404 |
|  | 0%,3g/L,6day |  |  |  |  |  |  |  | 120.9 | 4.217 |
|  | 35%,1g/L,2day |  |  |  |  |  |  |  | 118.9 | 4.404 |
|  | 35%,1g/L,4day |  |  |  |  |  |  |  | 117.8 | 4.217 |
|  | 35%,3g/L,2day |  |  |  |  |  |  |  | 117.7 | 4.217 |
|  | 55%,0g/L,2day |  |  |  |  |  |  |  | 117.6 | 4.869 |
|  | 0%,3g/L,4day |  |  |  |  |  |  |  | 117.4 | 4.217 |
|  | 55%,3g/L,4day |  |  |  |  |  |  |  | 116.5 | 4.619 |
|  | 55%,0g/L,4day |  |  |  |  |  |  |  | 115.1 | 4.404 |
|  | 35%,0g/L,2day |  |  |  |  |  |  |  | 114.7 | 4.869 |
|  | 0%,1g/L,6day |  |  |  |  |  |  |  | 114.2 | 4.869 |
|  | 55%,1g/L,4day |  |  |  |  |  |  |  | 113.2 | 4.619 |
|  | 35%,3g/L,6day |  |  |  |  |  |  |  | 112.7 | 4.869 |
|  | 0%,0g/L,6day |  |  |  |  |  |  |  | 112.4 | 4.404 |
|  | 55%,3g/L,6day |  |  |  |  |  |  |  | 110.6 | 4.217 |
|  | 35%,0g/L,4day |  |  |  |  |  |  |  | 108.6 | 4.869 |
|  | 55%,1g/L,2day |  |  |  |  |  |  |  | 107.2 | 4.404 |
|  | 0%,1g/L,4day |  |  |  |  |  |  |  | 101.0 | 4.619 |
|  | 55%,0g/L,6day |  |  |  |  |  |  |  | 97.5 | 4.217 |
|  | 35%,0g/L,6day |  |  |  |  |  |  |  | 86.55 | 4.404 |
| No. of Inflorescences per plant | 35%,3g/L,2day |  |  |  |  |  |  |  | 10.83 | 0.8184 |
|  | 0%,3g/L,2day |  |  |  |  |  |  |  | 10.64 | 0.8547 |
|  | 35%,3g/L,6day |  |  |  |  |  |  |  | 9.111 | 0.9450 |
|  | 35%,3g/L,4day |  |  |  |  |  |  |  | 8.750 | 0.8183 |
|  | 55%,1g/L,2day |  |  |  |  |  |  |  | 8.455 | 0.8547 |
|  | 35%,1g/L,2day |  |  |  |  |  |  |  | 8.455 | 0.8547 |
|  | 0%,3g/L,6day |  |  |  |  |  |  |  | 8.333 | 0.8184 |
|  | 35%,1g/L,6day |  |  |  |  |  |  |  | 7.750 | 0.8184 |
|  | 55%,3g/L,2day |  |  |  |  |  |  |  | 7.600 | 0.8965 |
|  | 55%,3g/L,6day |  |  |  |  |  |  |  | 7.167 | 0.8184 |
|  | 35%,1g/L,4day |  |  |  |  |  |  |  | 7.167 | 0.8184 |
|  | 0%,3g/L,4day |  |  |  |  |  |  |  | 6.833 | 0.8184 |
|  | 55%,1g/L,4day |  |  |  |  |  |  |  | 6.400 | 0.8965 |
|  | 55%,3g/L,4day |  |  |  |  |  |  |  | 6.000 | 0.8965 |
|  | 0%,1g/L,2day |  |  |  |  |  |  |  | 5.583 | 0.8184 |
|  | 55%,0g/L,6day |  |  |  |  |  |  |  | 5.417 | 0.8184 |
|  | 0%,1g/L,6day |  |  |  |  |  |  |  | 5.333 | 0.9450 |
|  | 0%,1g/L,4day |  |  |  |  |  |  |  | 5.300 | 0.8965 |
|  | 35%,0g/L,6day |  |  |  |  |  |  |  | 5.182 | 0.8547 |
|  | 55%,1g/L,6day |  |  |  |  |  |  |  | 4.917 | 0.8184 |
|  | 0%,0g/L,6day |  |  |  |  |  |  |  | 4.273 | 0.8547 |
|  | 0%,0g/L,2day |  |  |  |  |  |  |  | 4.083 | 0.8184 |
|  | 55%,0g/L,2day |  |  |  |  |  |  |  | 3.778 | 0.9450 |
|  | 35%,0g/L,2day |  |  |  |  |  |  |  | 3.556 | 0.9450 |
|  | 0%,0g/L,4day |  |  |  |  |  |  |  | 3.182 | 0.8547 |
|  | 55%,0g/L,4day |  |  |  |  |  |  |  | 2.818 | 0.8547 |
|  | 35%,0g/L,4day |  |  |  |  |  |  |  | 2.222 | 0.9450 |
| Inflorescence Length | 0%,1g/L,2day |  |  |  |  |  |  |  | 9.374 | 0.4952 |
|  | 0%,0g/L,2day |  |  |  |  |  |  |  | 7.354 | 0.4952 |
|  | 55%,3g/L,2day |  |  |  |  |  |  |  | 7.333 | 0.5425 |
|  | 0%,3g/L,2day |  |  |  |  |  |  |  | 6.515 | 0.5172 |
|  | 0%,1g/L,4day |  |  |  |  |  |  |  | 6.403 | 0.5425 |
|  | 35%,1g/L,2day |  |  |  |  |  |  |  | 6.121 | 0.5172 |
|  | 0%,1g/L,6day |  |  |  |  |  |  |  | 6.104 | 0.5718 |
|  | 55%,0g/L,2day |  |  |  |  |  |  |  | 6.046 | 0.5718 |
|  | 55%,1g/L,2day |  |  |  |  |  |  |  | 6.027 | 0.5172 |
|  | 55%,1g/L,6day |  |  |  |  |  |  |  | 5.938 | 0.4952 |
|  | 35%,0g/L,4day |  |  |  |  |  |  |  | 5.928 | 0.5718 |
|  | 35%,3g/L,2day |  |  |  |  |  |  |  | 5.878 | 0.4952 |
|  | 55%,3g/L,6day |  |  |  |  |  |  |  | 5.639 | 0.4952 |
|  | 0%,3g/L,4day |  |  |  |  |  |  |  | 5.558 | 0.4952 |
|  | 0%,3g/L,6day |  |  |  |  |  |  |  | 5.528 | 0.4952 |
|  | 55%,3g/L,4day |  |  |  |  |  |  |  | 5.463 | 0.5425 |
|  | 35%,1g/L,6day |  |  |  |  |  |  |  | 5.389 | 0.4952 |
|  | 35%,3g/L,4day |  |  |  |  |  |  |  | 5.372 | 0.4952 |
|  | 0%,0g/L,4day |  |  |  |  |  |  |  | 5.349 | 0.5172 |
|  | 0%,0g/L,6day |  |  |  |  |  |  |  | 5.311 | 0.5172 |
|  | 55%,0g/L,6day |  |  |  |  |  |  |  | 5.125 | 0.4952 |
|  | 55%,0g/L,4day |  |  |  |  |  |  |  | 5.077 | 0.5172 |
|  | 35%,3g/L,6day |  |  |  |  |  |  |  | 5.044 | 0.5718 |
|  | 35%,1g/L,4day |  |  |  |  |  |  |  | 5.028 | 0.4952 |
|  | 35%,0g/L,2day |  |  |  |  |  |  |  | 4.952 | 0.5718 |
|  | 55%,1g/L,4day |  |  |  |  |  |  |  | 4.657 | 0.5425 |
|  | 35%,0g/L,6day |  |  |  |  |  |  |  | 4.073 | 0.5172 |
| No. of Flowers per Inflorescence | 0%,1g/L,2day | a |  |  |  |  |  |  | 259.7 | 16.12 |
|  | 55%,3g/L,2day | a | b |  |  |  |  |  | 195.5 | 17.66 |
|  | 0%,0g/L,2day |  | b | c |  |  |  |  | 155.5 | 16.12 |
|  | 0%,1g/L,4day |  | b | c |  |  |  |  | 150.0 | 17.66 |
|  | 0%,3g/L,2day |  | b | c |  |  |  |  | 149.3 | 16.84 |
|  | 35%,1g/L,2day |  | b | c | d |  |  |  | 139.5 | 16.84 |
|  | 35%,3g/L,2day |  | b | c | d |  |  |  | 136.4 | 16.12 |
|  | 55%,3g/L,4day |  | b | c | d |  |  |  | 133.9 | 17.66 |
|  | 55%,0g/L,2day |  | b | c | d |  |  |  | 132.1 | 18.61 |
|  | 55%,3g/L,6day |  | b | c | d |  |  |  | 129.9 | 16.12 |
|  | 0%,3g/L,6day |  | b | c | d |  |  |  | 123.1 | 16.12 |
|  | 0%,0g/L,4day |  | b | c | d |  |  |  | 122.8 | 16.84 |
|  | 55%,1g/L,2day |  | b | c | d |  |  |  | 115.8 | 16.84 |
|  | 35%,0g/L,4day |  | b | c | d |  |  |  | 115.5 | 18.61 |
|  | 0%,0g/L,6day |  | b | c | d |  |  |  | 114.8 | 16.84 |
|  | 0%,3g/L,4day |  | b | c | d |  |  |  | 113.2 | 16.12 |
|  | 0%,1g/L,6day |  | b | c | d |  |  |  | 112.6 | 18.61 |
|  | 55%,1g/L,6day |  | b | c | d |  |  |  | 112.3 | 16.12 |
|  | 55%,0g/L,6day |  | b | c | d |  |  |  | 112.2 | 16.12 |
|  | 35%,1g/L,4day |  |  | c | d |  |  |  | 103.7 | 16.12 |
|  | 35%,3g/L,4day |  |  | c | d |  |  |  | 100.1 | 16.12 |
|  | 35%,0g/L,2day |  |  | c | d |  |  |  | 98.31 | 18.61 |
|  | 35%,1g/L,6day |  |  | c | d |  |  |  | 95.28 | 16.12 |
|  | 55%,0g/L,4day |  |  | c | d |  |  |  | 85.98 | 16.84 |
|  | 35%,3g/L,6day |  |  | c | d |  |  |  | 80.56 | 18.61 |
|  | 55%,1g/L,4day |  |  | c | d |  |  |  | 79.77 | 17.66 |
|  | 35%,0g/L,6day |  |  |  | d |  |  |  | 56.95 | 16.84 |
| No. of Flowers per plant | 55%,3g/L,2day |  |  |  |  |  |  |  | 1608 | 181.3 |
|  | 0%,3g/L,2day |  |  |  |  |  |  |  | 1555 | 172.9 |
|  | 0%,1g/L,2day |  |  |  |  |  |  |  | 1545 | 165.5 |
|  | 35%,3g/L,2day |  |  |  |  |  |  |  | 1532 | 165.5 |
|  | 35%,1g/L,2day |  |  |  |  |  |  |  | 1247 | 172.9 |
|  | 0%,3g/L,6day |  |  |  |  |  |  |  | 1068 | 165.5 |
|  | 55%,1g/L,2day |  |  |  |  |  |  |  | 1000 | 172.9 |
|  | 55%,3g/L,6day |  |  |  |  |  |  |  | 976.9 | 165.5 |
|  | 55%,3g/L,4day |  |  |  |  |  |  |  | 869.2 | 181.3 |
|  | 35%,1g/L,6day |  |  |  |  |  |  |  | 866.6 | 165.5 |
|  | 0%,1g/L,4day |  |  |  |  |  |  |  | 858.4 | 181.3 |
|  | 35%,3g/L,4day |  |  |  |  |  |  |  | 858.0 | 165.5 |
|  | 35%,3g/L,6day |  |  |  |  |  |  |  | 829.2 | 191.2 |
|  | 0%,3g/L,4day |  |  |  |  |  |  |  | 804.3 | 165.5 |
|  | 35%,1g/L,4day |  |  |  |  |  |  |  | 753.9 | 165.5 |
|  | 0%,0g/L,2day |  |  |  |  |  |  |  | 672.4 | 165.5 |
|  | 0%,1g/L,6day |  |  |  |  |  |  |  | 638.4 | 191.2 |
|  | 55%,0g/L,6day |  |  |  |  |  |  |  | 606.2 | 165.5 |
|  | 55%,1g/L,6day |  |  |  |  |  |  |  | 536.6 | 165.5 |
|  | 55%,0g/L,2day |  |  |  |  |  |  |  | 507.2 | 191.2 |
|  | 55%,1g/L,4day |  |  |  |  |  |  |  | 488.2 | 181.3 |
|  | 0%,0g/L,6day |  |  |  |  |  |  |  | 484.5 | 172.9 |
|  | 0%,0g/L,4day |  |  |  |  |  |  |  | 384.2 | 172.9 |
|  | 35%,0g/L,2day |  |  |  |  |  |  |  | 322.6 | 191.2 |
|  | 35%,0g/L,6day |  |  |  |  |  |  |  | 297.5 | 172.9 |
|  | 55%,0g/L,4day |  |  |  |  |  |  |  | 276.3 | 172.9 |
|  | 35%,0g/L,4day |  |  |  |  |  |  |  | 200.7 | 191.2 |
| Nectar volume per flower | 0%,0g/L,2day |  |  |  |  |  |  |  | 0.8708 | 0.0916 |
|  | 0%,0g/L,4day |  |  |  |  |  |  |  | 0.4354 | 0.0916 |
|  | 55%,1g/L,2day |  |  |  |  |  |  |  | 0.4296 | 0.0916 |
|  | 55%,1g/L,4day |  |  |  |  |  |  |  | 0.4238 | 0.0916 |
|  | 35%,0g/L,2day |  |  |  |  |  |  |  | 0.4196 | 0.0916 |
|  | 0%,1g/L,2day |  |  |  |  |  |  |  | 0.4150 | 0.1003 |
|  | 35%,1g/L,6day |  |  |  |  |  |  |  | 0.3988 | 0.0916 |
|  | 0%,0g/L,6day |  |  |  |  |  |  |  | 0.3979 | 0.0916 |
|  | 35%,1g/L,2day |  |  |  |  |  |  |  | 0.3910 | 0.1003 |
|  | 0%,1g/L,4day |  |  |  |  |  |  |  | 0.3854 | 0.0916 |
|  | 35%,3g/L,2day |  |  |  |  |  |  |  | 0.3850 | 0.1003 |
|  | 35%,0g/L,4day |  |  |  |  |  |  |  | 0.3842 | 0.0916 |
|  | 55%,0g/L,4day |  |  |  |  |  |  |  | 0.3742 | 0.0916 |
|  | 0%,1g/L,6day |  |  |  |  |  |  |  | 0.3729 | 0.0916 |
|  | 35%,0g/L,6day |  |  |  |  |  |  |  | 0.3696 | 0.0848 |
|  | 35%,1g/L,4day |  |  |  |  |  |  |  | 0.3675 | 0.0916 |
|  | 55%,0g/L,2day |  |  |  |  |  |  |  | 0.3554 | 0.0916 |
|  | 55%,3g/L,2day |  |  |  |  |  |  |  | 0.3425 | 0.0916 |
|  | 0%,3g/L,6day |  |  |  |  |  |  |  | 0.3313 | 0.0916 |
|  | 0%,3g/L,4day |  |  |  |  |  |  |  | 0.3208 | 0.0916 |
|  | 0%,3g/L,2day |  |  |  |  |  |  |  | 0.3108 | 0.0916 |
|  | 55%,0g/L,6day |  |  |  |  |  |  |  | 0.3108 | 0.0916 |
|  | 55%,1g/L,6day |  |  |  |  |  |  |  | 0.3075 | 0.0916 |
|  | 55%,3g/L,4day |  |  |  |  |  |  |  | 0.2946 | 0.0916 |
|  | 35%,3g/L,6day |  |  |  |  |  |  |  | 0.2829 | 0.0916 |
|  | 35%,3g/L,4day |  |  |  |  |  |  |  | 0.2342 | 0.0916 |
|  | 55%,3g/L,6day |  |  |  |  |  |  |  | 0.2246 | 0.0916 |
| Free sugar content (µg/µL) | 35%,3g/L,6day |  |  |  |  |  |  |  | 1,510.8 | 147.9 |
|  | 35%,0g/L,4day |  |  |  |  |  |  |  | 909.1 | 147.9 |
|  | 55%,3g/L,2day |  |  |  |  |  |  |  | 901.4 | 147.9 |
|  | 55%,3g/L,4day |  |  |  |  |  |  |  | 899.5 | 147.9 |
|  | 55%,0g/L,6day |  |  |  |  |  |  |  | 897.2 | 147.9 |
|  | 0%,0g/L,4day |  |  |  |  |  |  |  | 896.4 | 147.9 |
|  | 35%,1g/L,2day |  |  |  |  |  |  |  | 888.1 | 162.1 |
|  | 0%,1g/L,4day |  |  |  |  |  |  |  | 886.0 | 147.9 |
|  | 0%,1g/L,2day |  |  |  |  |  |  |  | 880.8 | 162.1 |
|  | 0%,3g/L,4day |  |  |  |  |  |  |  | 859.6 | 147.9 |
|  | 0%,3g/L,2day |  |  |  |  |  |  |  | 852.9 | 147.9 |
|  | 55%,1g/L,2day |  |  |  |  |  |  |  | 850.4 | 147.9 |
|  | 35%,0g/L,6day |  |  |  |  |  |  |  | 841.8 | 137.0 |
|  | 55%,1g/L,6day |  |  |  |  |  |  |  | 836.8 | 147.9 |
|  | 0%,3g/L,6day |  |  |  |  |  |  |  | 832.2 | 147.9 |
|  | 35%,1g/L,4day |  |  |  |  |  |  |  | 808.9 | 147.9 |
|  | 35%,1g/L,6day |  |  |  |  |  |  |  | 808.7 | 147.9 |
|  | 35%,0g/L,2day |  |  |  |  |  |  |  | 802.2 | 147.9 |
|  | 35%,3g/L,4day |  |  |  |  |  |  |  | 795.6 | 147.9 |
|  | 55%,1g/L,4day |  |  |  |  |  |  |  | 789.0 | 147.9 |
|  | 55%,0g/L,4day |  |  |  |  |  |  |  | 770.3 | 147.9 |
|  | 0%,1g/L,6day |  |  |  |  |  |  |  | 746.1 | 147.9 |
|  | 0%,0g/L,6day |  |  |  |  |  |  |  | 727.9 | 147.9 |
|  | 35%,3g/L,2day |  |  |  |  |  |  |  | 710.8 | 162.1 |
|  | 55%,0g/L,2day |  |  |  |  |  |  |  | 665.9 | 147.9 |
|  | 0%,0g/L,2day |  |  |  |  |  |  |  | 625.7 | 147.9 |
|  | 55%,3g/L,6day |  |  |  |  |  |  |  | 589.0 | 147.9 |
